# From Organic Fragments to Photoswitchable Catalysts: The OFF–ON Structural Repository for Transferable Kernel-Based Potentials

**DOI:** 10.1021/acs.jcim.3c01953

**Published:** 2024-02-06

**Authors:** Frédéric Célerse, Matthew D. Wodrich, Sergi Vela, Simone Gallarati, Raimon Fabregat, Veronika Juraskova, Clémence Corminboeuf

**Affiliations:** † Laboratory for Computational Molecular Design (LCMD), Institute of Chemical Sciences and Engineering, Ecole Polytechnique Fédérale de Lausanne (EPFL), Lausanne 1015, Switzerland; ‡ National Center for Competence in Research-Catalysis (NCCR-Catalysis), Ecole Polytechnique Fédérale de Lausanne, Lausanne 1015, Switzerland; § National Centre for Computational Design and Discovery of Novel Materials (MARVEL), Ecole Polytechnique Fédérale de Lausanne, Lausanne 1015, Switzerland

## Abstract

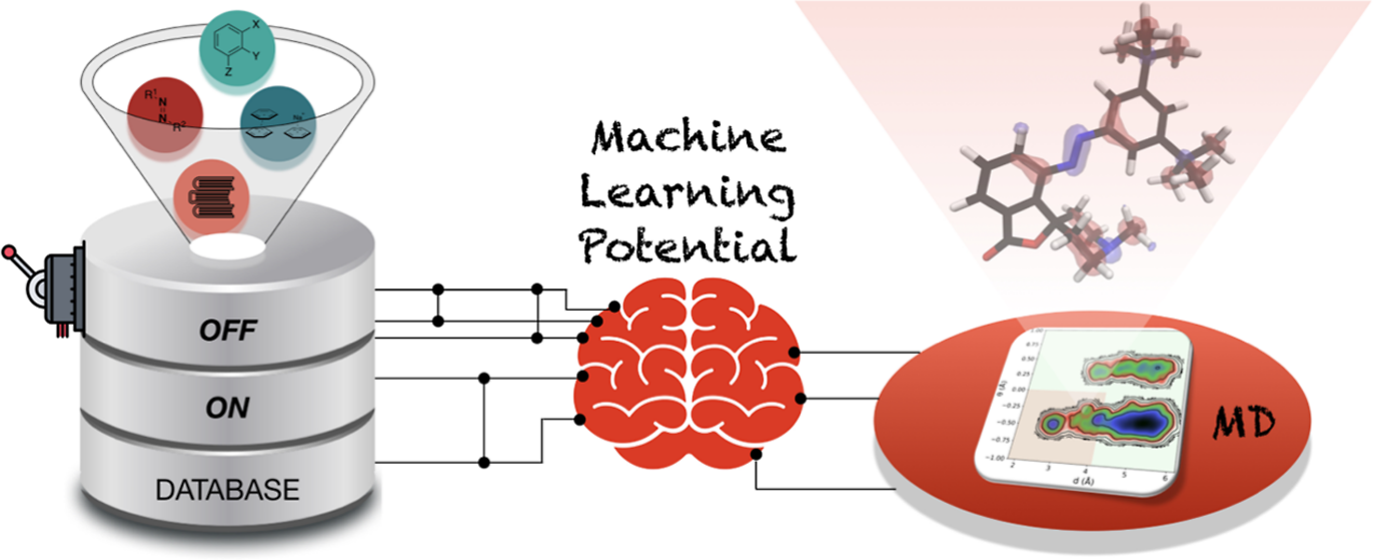

Structurally and conformationally diverse databases are needed to train accurate neural networks or kernel-based potentials capable of exploring the complex free energy landscape of flexible functional organic molecules. Curating such databases for species beyond “simple” drug-like compounds or molecules composed of well-defined building blocks (e.g., peptides) is challenging as it requires thorough chemical space mapping and evaluation of both chemical and conformational diversities. Here, we introduce the OFF–ON (organic fragments from organocatalysts that are non-modular) database, a repository of 7869 equilibrium and 67,457 nonequilibrium geometries of organic compounds and dimers aimed at describing conformationally flexible functional organic molecules, with an emphasis on photoswitchable organocatalysts. The relevance of this database is then demonstrated by training a local kernel regression model on a low-cost semiempirical baseline and comparing it with a PBE0-D3 reference for several known catalysts, notably the free energy surfaces of exemplary photoswitchable organocatalysts. Our results demonstrate that the OFF–ON data set offers reliable predictions for simulating the conformational behavior of virtually any (photoswitchable) organocatalyst or organic compound composed of H, C, N, O, F, and S atoms, thereby opening a computationally feasible route to explore complex free energy surfaces in order to rationalize and predict catalytic behavior.

## 1 Introduction

Machine learning (ML)-based potentials have served as efficient ab initio surrogates for molecular dynamics (MD) simulations, enabling statistically converged free energy surfaces (FESs) to be computed at DFT-level accuracy with significantly reduced computational cost.^1−24^ Commonly used architectures include artificial neural networks (ANN),^25−31^ Gaussian approximation models (GAP),^32−35^ and, more generally, kernel-based methods.^36−42^ The success and robustness of these surrogate potentials rely on many aspects, with the curation of chemically and structurally diverse databases to train the underlying ML models being of utmost importance for achieving size-extensive extrapolations and transferable predictions. Generating such training sets requires not only a thorough mapping of chemical space but also the inclusion of out-of-equilibrium structures that cover the necessary energies and forces for MD simulations. The majority of existing databases include equilibrium structures aimed at describing static properties (e.g., HOMO–LUMO energies, polarizabilities, and dipole moments) of finite classes of molecules, such as “small” drug-like compounds typically composed of 10–20 heavy atoms^43−49^ or larger systems with reduced chemical diversity, such as peptides composed of the same amino acids.^50−52^ The largest of these data sets is GDB-17,^43^ which includes 166.4 billion species totaling up to 17 C, N, O, S, and halogen atoms.

Recently, numerous research groups have created databases suitable for training ML potentials for MD simulations that include out-of-equilibrium structures.^26,53−58^ Notably, these include works from Roitberg et al. (20 M out-of-equilibrium conformations for 57,462 organic molecules with broad chemical diversity),^26^ Miller et al. (2.3 M molecules including 20k salts and non-covalently bound complexes in different protonation and tautomeric states),^53^ Barbatti et al. (1.2 M equilibrium and non-equilibrium geometries of 10 flexible organic molecules),^54^ and Eastman et al. (1.1 M conformations of small drug-like molecules, dimers, dipeptides, and solvated amino acids).^55^ Nonetheless, attaining a “fully general” database that could be used to train models for MD simulations on any molecular system remains nonpragmatic for myriad reasons (e.g., database size and expense of reference computations). Instead, the current state-of-the-art is to train databases tailored to specific chemical problems.

Despite the inherent difficulties, creating a more general database that could be used to train ML potentials to describe, for example, the chemical reactivity and the rich conformational and configurational behavior of flexible medium- and large-sized functional organic molecules would be highly valuable. Particularly, it is not only the unique chemical components but also the dynamic movements of these organic molecules that are intrinsically linked to their functionality (e.g., the flexible nature of organocatalysts influences both selectivity and reactivity).^59,60^ Such problems are particularly well illustrated by photoswitchable organocatalysts (i.e., photochromic molecules featuring a catalytic site that can be toggled between two stereoelectronic states with different reactivities and flexibilities when acted upon by appropriate wavelengths of light).^61−71^ While data-driven discovery pipelines exist to engineer molecular photoswitches with desired photophysical properties,^72−75^ fewer studies have focused on investigating chemical reactivity as a function of the rich conformational and configurational behavior of these organocatalysts. Add to this the requirement that each configurational state [e.g., (*E*)- or (*Z*)-isomer] uniquely corresponds to an “ON” or “OFF” reactivity state,^76^ and it becomes clear that a thorough description of conformational states is needed to fully grasp the catalytic properties. In such cases, accurate MD simulations with enhanced sampling techniques are necessary since the energetic ordering of conformations is dictated by the subtle interplay between full entropic and anharmonic contributions, noncovalent interactions, and environmental effects.

In contrast to the existing databases used to train ML models for MD simulations (vide supra), the principal problem with constructing a suitable database for functional organic molecules is their lack of obvious “modularity”.^77,78^ No unique set of components exists from which they are constructed, in contrast to, for example, how polypeptides are constructed from amino acid residues or organometallic complexes from metal centers and ligands. Here, we propose a strategy for systematically constructing conformationally and chemically diverse databases for molecular systems that lack such modularity with emphasis placed on photoswitchable organocatalysts. To accomplish this, we selected organocatalysts from the literature and fragmented them into smaller components (see Section 2 and the Supporting Information for further information). In this way, sufficient chemical diversity is ensured to facilitate the description of the free energies of inherently nonmodular organocatalytic systems. The newly created OFF–ON (organic fragments from organocatalysts that are non-modular) repository can be used to describe the conformational behavior of chemically diverse functional organic molecules, including those possessing photoswitching units and catalytic motifs.

Here, we leverage the OFF–ON database along with our recently reported local kernel regression (LKR) framework with an orthogonal matching pursuit algorithm (LKR–OMP^79^) to create accurate potential energy surfaces of functional organic molecules from inexpensive baseline computations. Specifically, we predict the energetic correction between a semiempirical density functional tight-binding (DFTB) baseline and the PBE0-D3 energies for a set of relaxed and out-of-equilibrium photoswitchable organocatalysts.^80^ The LKR–OMP algorithm, a specific local kernel-based method, provides the benefit of requiring a smaller database and reduced training cost compared with, for example, Behler–Parrinello Neural Networks (BPNN). Subsequently, the MD simulations are more stable and reliable, which allow for FESs approaching chemical accuracy to be created.

## 2 Data Set Curation

**Figure 1:**
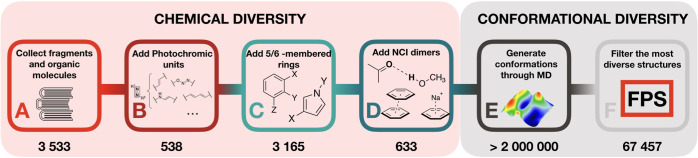
Workflow describing database curation. The number of structures generated at each step is depicted under each panel.

Our strategy for constructing a database aimed at covering the conformationally and configurationally diverse profiles of functional organic molecules is outlined in Figure 1. The process consists of two primary stages: first, curating a database encompassing a sufficient number of structures to represent the distinct chemical environments present in functional organic molecules and photoswitchable organocatalysts (e.g., ensuring chemical diversity; pink, Figure 1). Second, generating an array of conformers for each of these database structures to encompass out-of-equilibrium effects, which serves as the cornerstone for performing MD simulations (e.g., ensuring conformational diversity; gray, Figure 1).

### 2.1 Chemical Diversity

**Figure 2:**
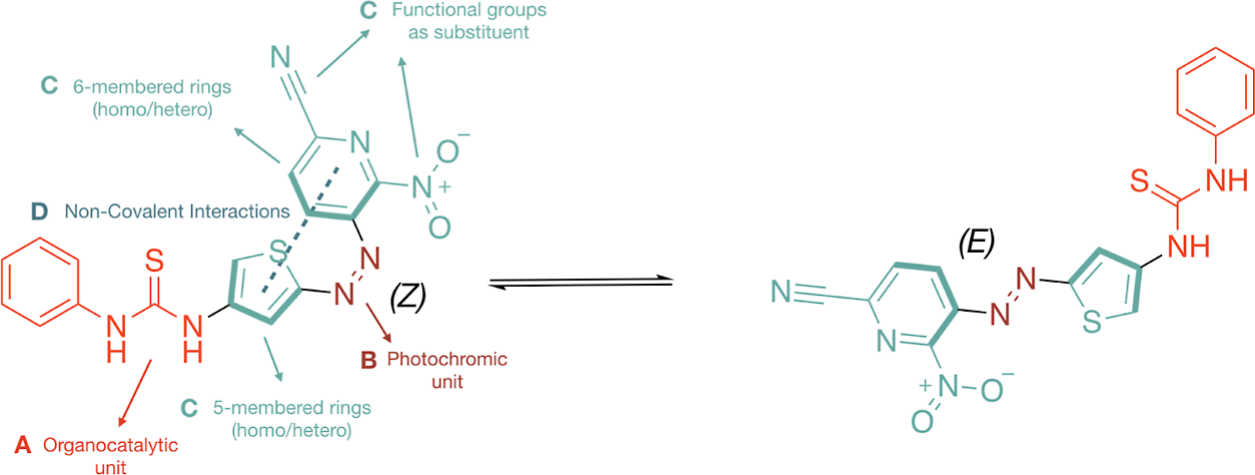
Schematic representation of a photoswitchable organocatalyst with different structural environments highlighted: (A) organocatalytic unit, (B) photochromic unit, (C) rings and ring substituents, and (D) non-covalent interactions.

The first step in constructing our database (cf. Figure 1A–D) involves generating a set of molecules that capture the diverse chemical environments specific to functional organic molecules (with emphasis herein on photoswitchable organocatalysts), as illustrated in Figure 2. These include the actual organocatalytic moieties (Figure 1A), the photochromic unit (responsible for interconverting between (*E*)- and (*Z*)-isomers) (Figure 1B), substituted aromatic subunits that link the photochromic and catalytic units (Figure 1C), and non-covalent interactions that may be present (Figure 1D). To accomplish this, organic molecules representative of the four chemical environments were either extracted from existing databases (e.g., OSCAR,^77^ CSD,^81^ PubChem,^47^ and NCI Atlas^82−84^) or generated (see Supporting Information Section 1, Tables S2 and S3 for further details). The pipeline adopted for generating and selecting each fragment molecule is reported in Supporting Information Sections 1.1–1.4. Broadly speaking, the primary consideration here is balancing the number of structures in each of the four classes in order to provide a consistent description for each of the four chemical environments. After selecting structures that ensure chemical diversity (particularly for 1A, see Supporting Information Section 1 for the detailed protocol used), we arrive at a total of 7869 entries (3533 catalytic moieties, 538 photochromic units, 3165 substituted rings, and 633 dimers representing non-covalent interactions).

### 2.2 Conformational Diversity

As our primary objective is to exploit the database (developed herein) for training ML models capable of describing the free energy landscapes of flexible and nonmodular photoswitchable organocatalysts, it is necessary to introduce multiple molecular conformations, including out-of-equilibrium structures. To accomplish this, 5 ps MD trajectories beginning from DFTB-optimized structures were run for each of the 7869 molecules obtained from the procedure outlined in Section 2.1 (Figure 1E). Naturally, this led to a massive number of new geometries (>2,000,000), which required further pruning to construct a chemically and structurally diverse database of reasonable size. Therefore, we extracted the most diverse conformations from each of the 7869 MD trajectories using the farthest point sampling (FPS) algorithm (see Supporting Information Section 1.5 for further details) to arrive at a total of 67,457 new out-of-equilibrium structures, Figure 1F.

### 2.3 “OFF–ON” Repository

Combining the two sets of structures detailed in Sections 2.1 and 2.2 gives a total of 75,326 structures that include both equilibrium and non-equilibrium states. This repository, dubbed “OFF–ON”, represents the chemical and conformational diversities of virtually any functional organic molecule and photoswitchable organocatalyst composed of only H, C, N, O, F, and S atoms. By including the most prevalent functional groups (see Supporting Information Table S1), noncovalent interactions, and photoswitching units, OFF–ON is tailored for training a LKR–OMP potential capable of providing FESs of flexible and nonmodular photoswitchable organocatalysts.

## 3 Computational Methods

### 3.1 Electronic Structure Computations

The baseline energy was computed at the DFTB3 level with 3ob parameters^85,86^ and D3 dispersion correction, as implemented in DFTB+^87^ and normalized using a multilinear regression model. The reference energy corresponds to the PBE0^88,89^ -D3^90^ /def2-SVP^91^ level obtained using the TeraChem code^92^ and normalized in the same manner as the baseline. The Δ-correction was then computed for each geometry as the difference between the normalized PBE0 and the DFTB energies.

### 3.2 ML Potentials: Sparse Local Kernel Regression

The LKR model combined with the Faber–Christensen–Huang–Lilienfeld (FCHL19) representation^93^ was used to target the difference between atomization energies computed at the DFTB-D3 level^94^ and PBE0-D3/def2-SVP reference, following the procedure of Fabregat et al.^79^ In line with our previous work,^79^ sparse LKR trained on the most relevant local atomic environments selected with the orthogonal matching pursuit (OMP) algorithm^95^ was used. Proceeding in this manner bypasses the tedious process of properly choosing the best atomic environments to optimize the energy predictions as the OMP selects the best set of atomic environments for each atom type based on those available within the database. Thus, LKR–OMP possesses the transferability and the scalability of neural networks while benefiting from the additional stability and reduced training cost of kernel-based methods.^79^

An initial pool of 40,000 environments was selected with the farthest point sampling (FPS) algorithm^96^ (i.e., 5000 for H, 12,000 for C, 8000 for N, 8000 for O, 2000 for F, and 5000 for S). Afterward, 1000 local atomic environments were selected by the OMP [i.e., OMP(1000)] to minimize the prediction error. The FCHL19 representation was generated with the QML-toolkit^97^ with a radial cutoff set to 5 Å and an angular cutoff set to 4 Å. For the kernel ridge, the width σ of the Gaussian kernel was set to 3.

### 3.3 Enhanced Sampling

Out-of-equilibrium structures were generated by temperature replica exchange simulations using the REMD@DFTB3 protocol implemented in the i-PI code.^98^ REMD was performed with eight replicas, with temperatures ranging from 300 to 800 K on a logarithmic scale. The equations of motion were integrated with a time step of 0.5 fs. A Langevin thermostat was used to control the temperature of the different replicas. Simulations were propagated for 2 ns to ensure comprehensive sampling of the conformational space.^99^ FPS was then applied to the Spectral London Axilrod–Teller–Muto (SLATM)^100^ representations of the geometries to arrive at a final pool of structures that was used as a test set for assessing the accuracy of the LKR–OMP correction (vide infra). Note that the SLATM representation was used here rather than FCHL19 as a global representation of the molecules sufficiently captures conformational diversity (since the FPS was used to select structures from the same MD simulations).

The FES of the photoswitches was computed via reservoir–Hamiltonian replica exchange (resH–RE) Monte Carlo, a variant of the temperature replica exchange method.^101^ Akin to our previous work, the system replicas were sampled in parallel using different Hamiltonians, which were constructed as a combination of a baseline *V*_baseline_ (DFTB) and an ML correction *V*_target_ as *V* = (1 – λ)*V*_target_ + λ*V*_baseline_, where λ is a factor going from 0 to 1. The replica with λ = 0 corresponds to the target level of theory. The last replica (i.e., λ = 1) was replaced by a canonical reservoir generated using the baseline to further facilitate the sampling.

The resH–RE simulations were run using the in-house Python package “MORESIM”,^101^ available on GitHub. Six replicas were used with the potential spaced linearly from DFTB to DFTB + LKR. The converged canonical reservoir was taken from the REMD@DFTB3 simulations in ref (76). All resH–RE simulations were run for 20 million Monte Carlo steps with the possibility of exchanging replicas every 20 steps. The Monte Carlo steps were guided using a global random displacement with a Gaussian distribution with a standard deviation equal to 0.001 Å. The acceptance rate of the simulations was 50%. The simulations were stopped once convergence of the free energy was reached.

## 4 Results and Discussion

### 4.1 Accuracy of the ML Potential

Our primary objective is to obtain the FES of any large and flexible organic molecule at a hybrid DFT level of theory while simultaneously circumventing the computational cost imposed by ab initio MD. To address this, we choose to use the in-house developed LKR–OMP model, which allows a robust and accurate machine-learning potential (MLP) to be developed with reduced training time compared to conventional neural network potentials (i.e., Behler–Parrinello-type neural networks).^79^ Subsequent analysis focuses on accomplishing this objective.

**Figure 3:**
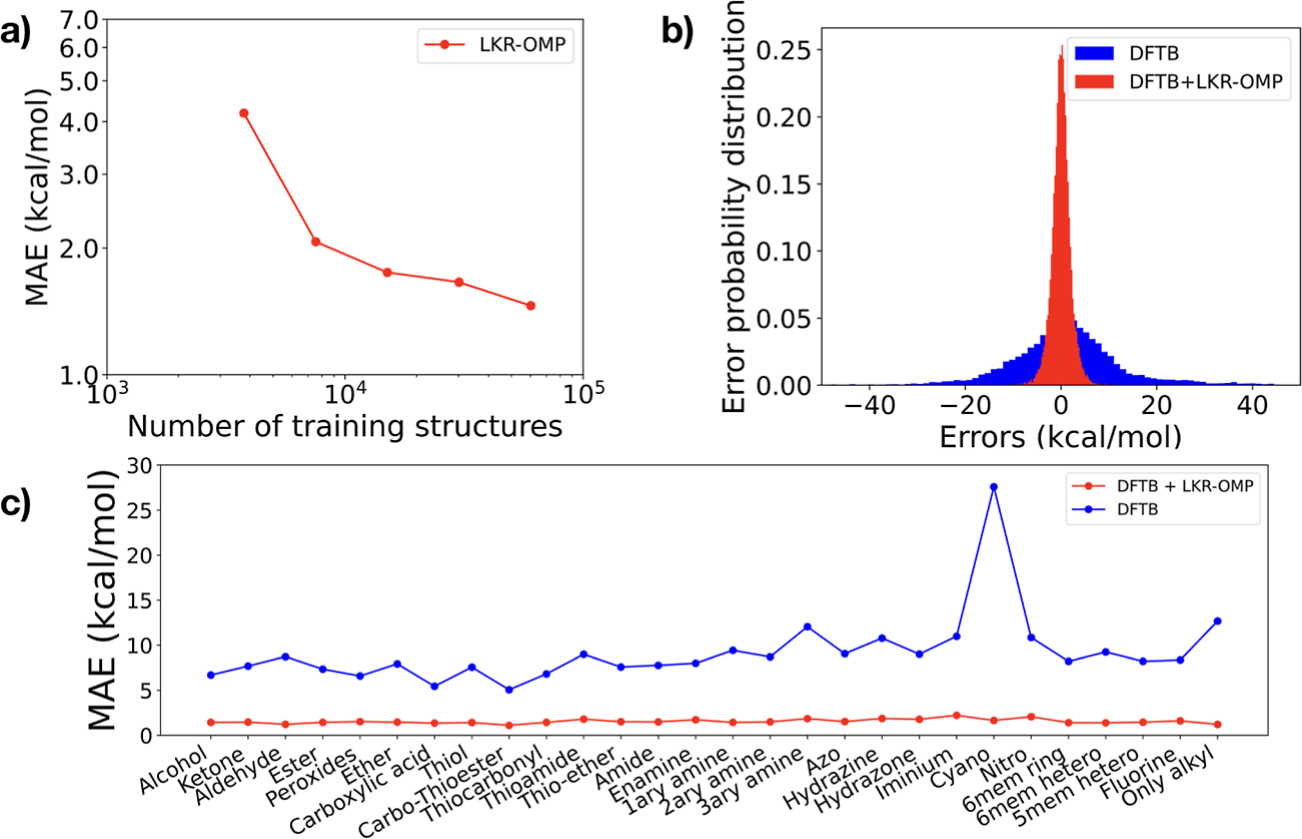
(a) Learning curve associated with MAE vs number of training structures. (b) Histogram of errors comparing DFTB with the DFTB + LKR correction trained on an 80/20 split. (c) MAE predicted for different functional groups using DFTB (blue) and the DFTB + LKR correction (red) with respect to the PBE0-D3 reference.

Utilizing the OFF–ON database, we trained an LKR–OMP model aimed at achieving PBE0-D3/def2-SVP-level accuracy from a DFTB energy baseline. The learning curve of the MLP is presented in Figure 3A. Using an 80/20 training/test split of the database (i.e., 60,260/15,066) that keeps the test set fixed while taking an increasing number of structures from the training set, we arrive at an error of 1.43 kcal/mol for the test set. The necessity of correcting the DFTB energies becomes clear when comparing the regression slopes and MAEs with and without the LKR–OMP corrections trained on the same 80/20 split (Figure 3B and Supporting Information Figure S1). While the original DFTB energies do not correlate well with the DFT reference data (MAE = 8.97 kcal/mol), the LKR–OMP-corrected energies show significant improvement (MAE = 1.46 kcal/mol). A more detailed analysis across different functional groups present in organocatalysts shows consistent improvement by the LKR–OMP correction (Figure 3C and Table S4 in Supporting Information), such that predictions can be made on highly complex and diverse data sets and the desired FESs can be reliably constructed.^79^

### 4.2 Extrapolation

**Figure 4:**
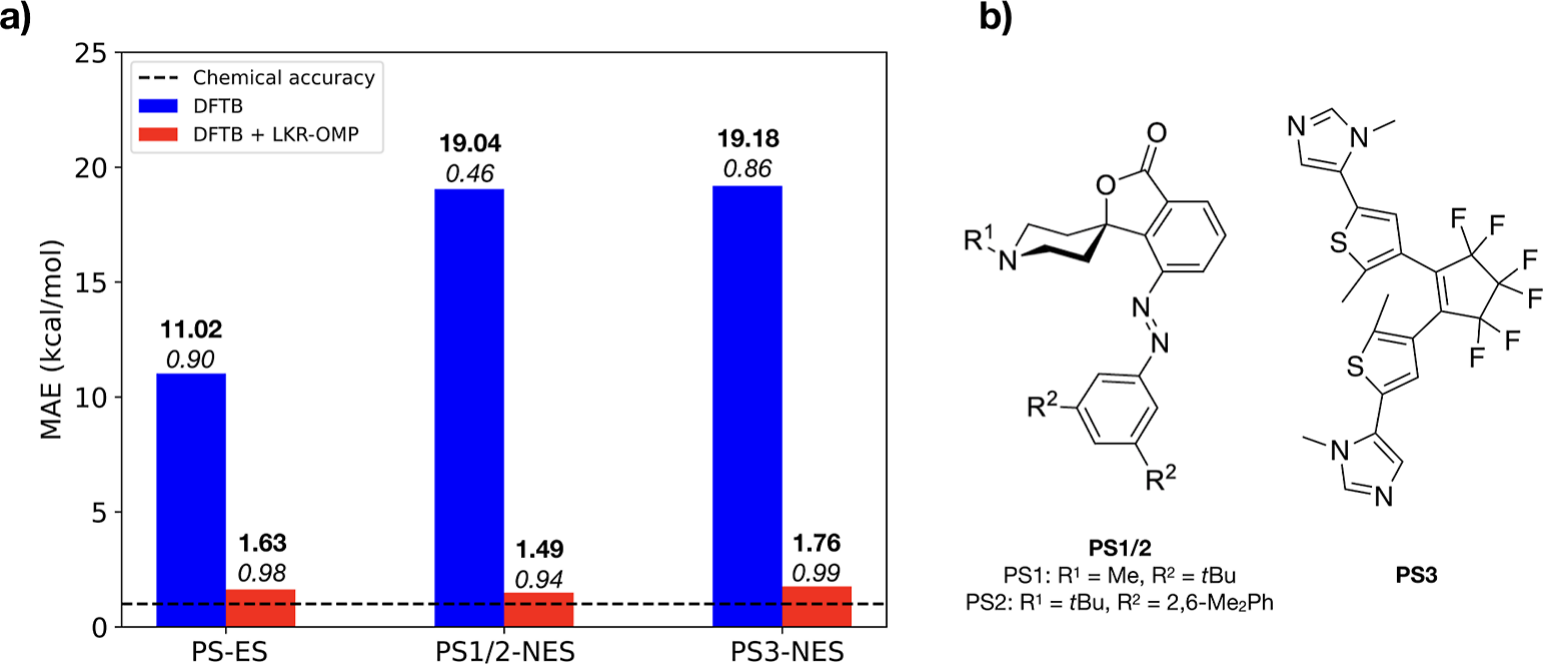
(a) Mean absolute error values on the energy prediction using DFTB (blue) and DFTB + LKR (red) relative to a PBE0-D3 reference for the equilibrium structures of 344 photoswitches (PS-ES) and nonequilibrium structures of PS1/PS2 (PS1-NES/PS2-NES) as well as PS3 (PS3-NES). (b) Structures of PS1–3. The absolute MAE values and their related *R*^2^ for each set of values are depicted in bold and italics, respectively.

To ensure that the OFF–ON database provides broad coverage of the chemical and conformational space, we next assessed the ability of the trained LKR–OMP model to obtain accurate energies of both equilibrium and nonequilibrium structures for a series of out-of-sample photoswitches and organocatalysts. Specifically, we examine the equilibrium structures (ES) of 344 photoswitches taken from the work of Griffiths et al.^72^ (PS-ES, Figure 4), along with non-equilibrium structures (NES) obtained from REMD@DFTB3^98^ (i.e., replica exchange MD employed with the DFTB-D3/3ob level of theory) snapshots of two photoswitchable organocatalysts: N-alkylated azobenzene-tethered piperidines in both the (*E*)- and (*Z*)-configuration (i.e., the “OFF” and “ON” configurations of PS1/2-NES,^102^Figure 4) and a dithienylethene-linked imidazole^103^ (PS3-NES, Figure 4). The equilibrium geometries of the 344 photoswitches were obtained by optimization at the DFTB-D3 level, while the structures of the two photoswitchable organocatalysts were selected from a 2 ns REMD@DFTB3 simulation performed at the DFTB-D3 level of theory (see Computational Methods).

While the DFTB and DFTB-LKR energies of the 344 photoswitch equilibrium structures (PS-ES set) each correlate well with the DFT reference values (*R*^2^ of 0.90 and 0.98, respectively, Figure 4a), the LKR–OMP correction considerably reduces the DFTB MAE (from 11.02 to 1.63 kcal/mol). The LKR–OMP correction also shows the ability to correct nonequilibrium conformations, reducing the MAE from 19 to 2 kcal/mol for both PS1/2-NES and PS3-NES while greatly improving the regression slopes (to 0.94 and 0.99 from 0.46 and 0.86, respectively, Figure 4a). These results are clear evidence that the LKR–OMP model is capable of extrapolating to larger molecules by reconstructing the energy of the complete structures from their components.

**Figure 5:**
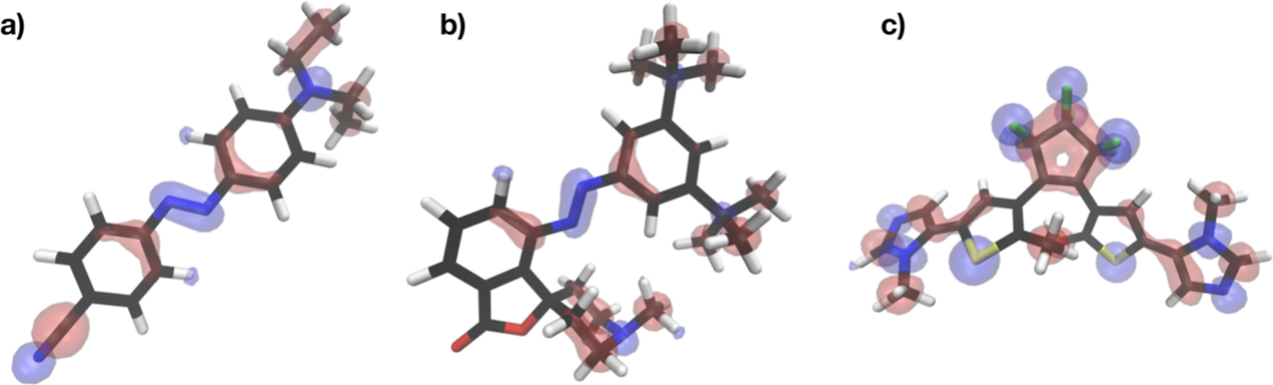
Representation of the LKR–OMP corrections on three independent structures extracted from the (a) PS–ES database, (b) PS1/2–NES, and (c) PS3–NES. The LKR–OMP corrections are shown using isosurfaces derived from a scalar field. This field was generated with the LKR–OMP atomic corrections to the energy and convoluted with the atomic positions and a Gaussian filter of width 1 Å. The isosurfaces correspond to the isovalues −10 and +10, represented in blue and red clouds, respectively. Hydrogen atoms are represented in white, carbon in black, nitrogen in blue, oxygen in red, fluorine in green, and sulfur in yellow.

The nature of the local ML correction provided by the LKR–OMP model also enables the visualization of molecular regions where the correction acts. In Figure 5, we construct a scalar field using atomic-centered Gaussian functions scaled to match the LKR–OMP atomic predictions^79^ for one of the 344 PS-ES photoswitches (Figure 5a), as well as for a single representative conformation taken from the full PS1/PS2 (Figure 5b) and the PS3 (Figure 5c) pool of structures (see Supporting Information Section 3 and Figure S2 for technical details). The results reveal a negative energetic LKR–OMP correction (a region where the DFTB is destabilized compared to that of the reference PBE0-D3 level, depicted in blue) that predominantly affects heteroatoms (Figure 5a,b and Supporting Information Figure S2). While oxygen seems to be well described, considerable discrepancies exist for nitrogen, sulfur, and fluorine atoms. Correspondingly, the carbon atoms bound to these problematic heteroatoms are characterized by positive energetic LKR–OMP contributions (a region where the DFTB is overly stabilized compared to that of the reference PBE0-D3 level, depicted in red). Taken together, the correction induces a smooth polarization between hetero and carbon atoms, particularly in the azo moiety, ring structures, and cyano-functional groups.

Generally speaking, our LKR–OMP correction emphasizes the limitations of DFTB to fully capture polarization effects within covalent bonds, an observation in agreement with Fabregat et al.’s^79^ finding regarding LKR–OMP’s capability to ensure an equally accurate description across all molecular regions. These examples demonstrate the ability of the LKR–OMP model to effectively predict accurate DFT energies for both equilibrium and out-of-equilibrium structures.

### 4.3 Case Study: The FES of a Photoswitchable Brønsted Base

When combined with accelerated sampling techniques, the LKR–OMP potential trained on the OFF–ON repository provides access to thorough FESs of virtually any flexible (photoswitchable) organocatalyst or other complex nonmodular organic systems composed of H, C, N, O, F, and S atoms. To demonstrate the power of this approach, we revisit the conformational behavior of two N-alkylated azobenzene-tethered piperidine photoswitches (PS1 and PS2, Figure 4), which catalyze the Henry reaction of nitroalkanes and aldehydes.^102,104^ The steric shielding of the piperidine lone pair and, therefore, the catalytic activity are controlled by the light-driven (*E*)-to-(*Z*)-isomerization process, with the (*Z*)-isomer being the catalytically active species (i.e., the “ON” state). As recently shown by our group,^76^ the lack of highly structured configurations decreases the effectiveness of the steric shielding strategy. This leads to a population of false ON and false OFF states in both the (*E*)- and (*Z*)-configurations that lead to activation/deactivation of the catalyst, even without a formal configurational change. Although the key findings related to the conformational behavior of the photoswitchable catalysts, such as the relative population of active or inactive states (corroborated by comparison to experimentally reported activity trends), remain valid, our previously published analysis was performed at a fairly low electronic structure level (REMD@DFTB3).^76^ Leveraging the LKR–OMP ML potential with resH–RE, we now map the FES of these photoswitches at the PBE0-D3 level of theory (see Supporting Information Figures S3 and S6 for further details on convergence).

**Figure 6:**
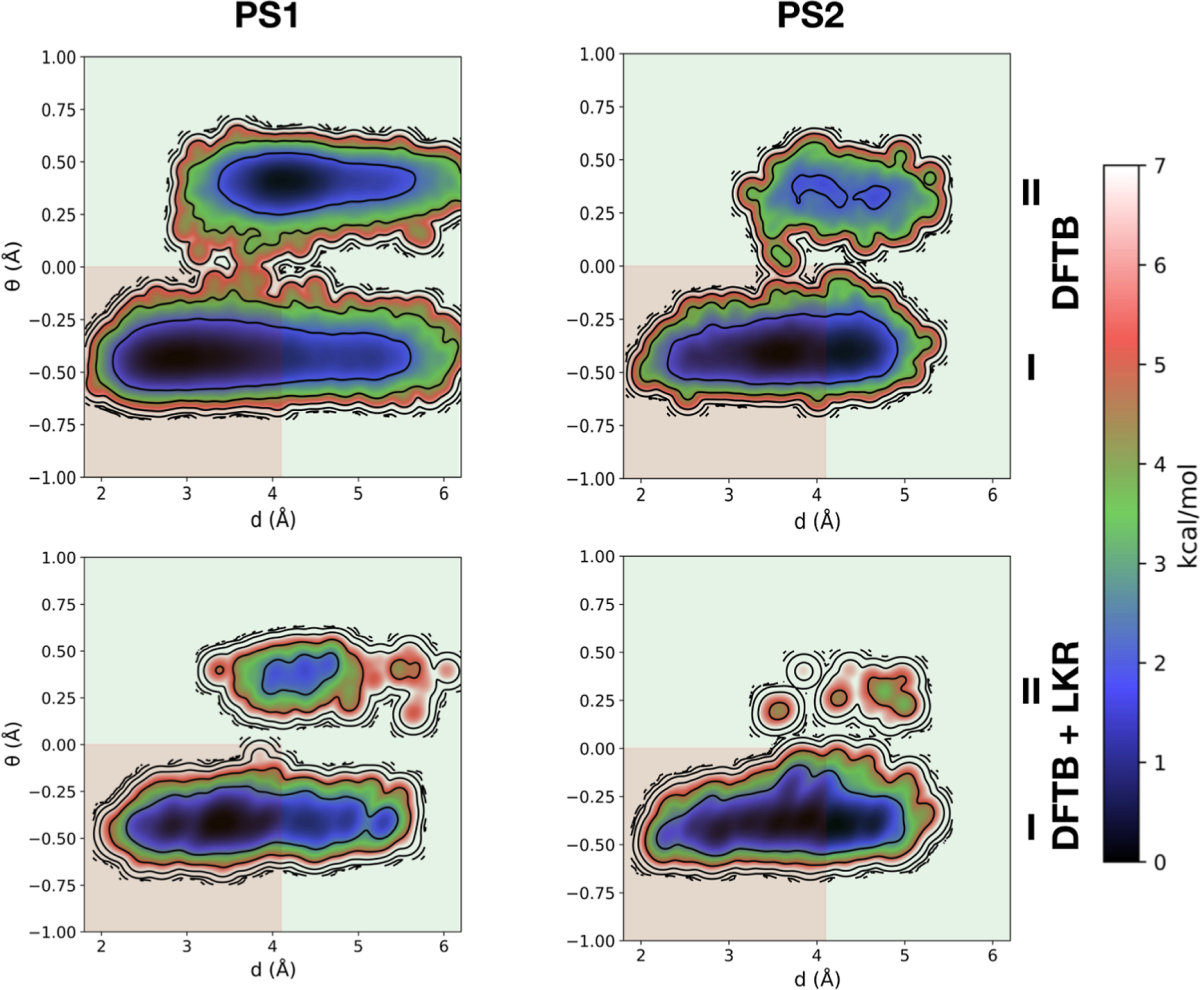
Computed FESs of (*E*)-PS1/2 at the DFTB level with and without the LKR–OMP correction. The background colors correspond to the “OFF” [red, where the nitrogen atom lone pair (i.e., the active site) is sterically shielded] and “ON” (green, active site accessible to the substrate) states of the catalyst.

**Scheme 1:**
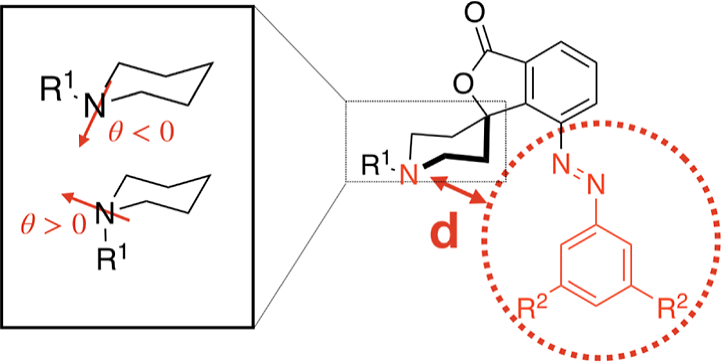
Collective Variables *d* and θ Chosen for PS1/2. PS1: R^1^ = Me, R^2^ = ^*t*^Bu. PS2: R^1^ = ^*t*^Bu, and R^2^ = 2,6-Me_2_Ph

Figure 6 depicts the FESs of PS1/2 represented in terms of two collective variables, *d* and θ (Scheme 1). The former is the shortest distance between the nitrogen atom of the piperidine ring and any other atom of the phenylazo group, while the latter is the distance between the piperidine’s N atom and the plane passing through the three carbon atoms to which the nitrogen is bound (R^1^–C and two C atoms of the piperidine ring). The conformational region of the FES (Figure 6) with θ < 0 is labeled **I** while that with θ > 0 is labeled **II**, represented by the upper and lower regions on the *y*-axis, respectively. In addition, the background colors correspond to the “OFF” [red, where the nitrogen atom lone pair (i.e., the active site) is sterically shielded] and “ON” (green, active site accessible to the substrate) states of the catalyst.

**Scheme 2:**
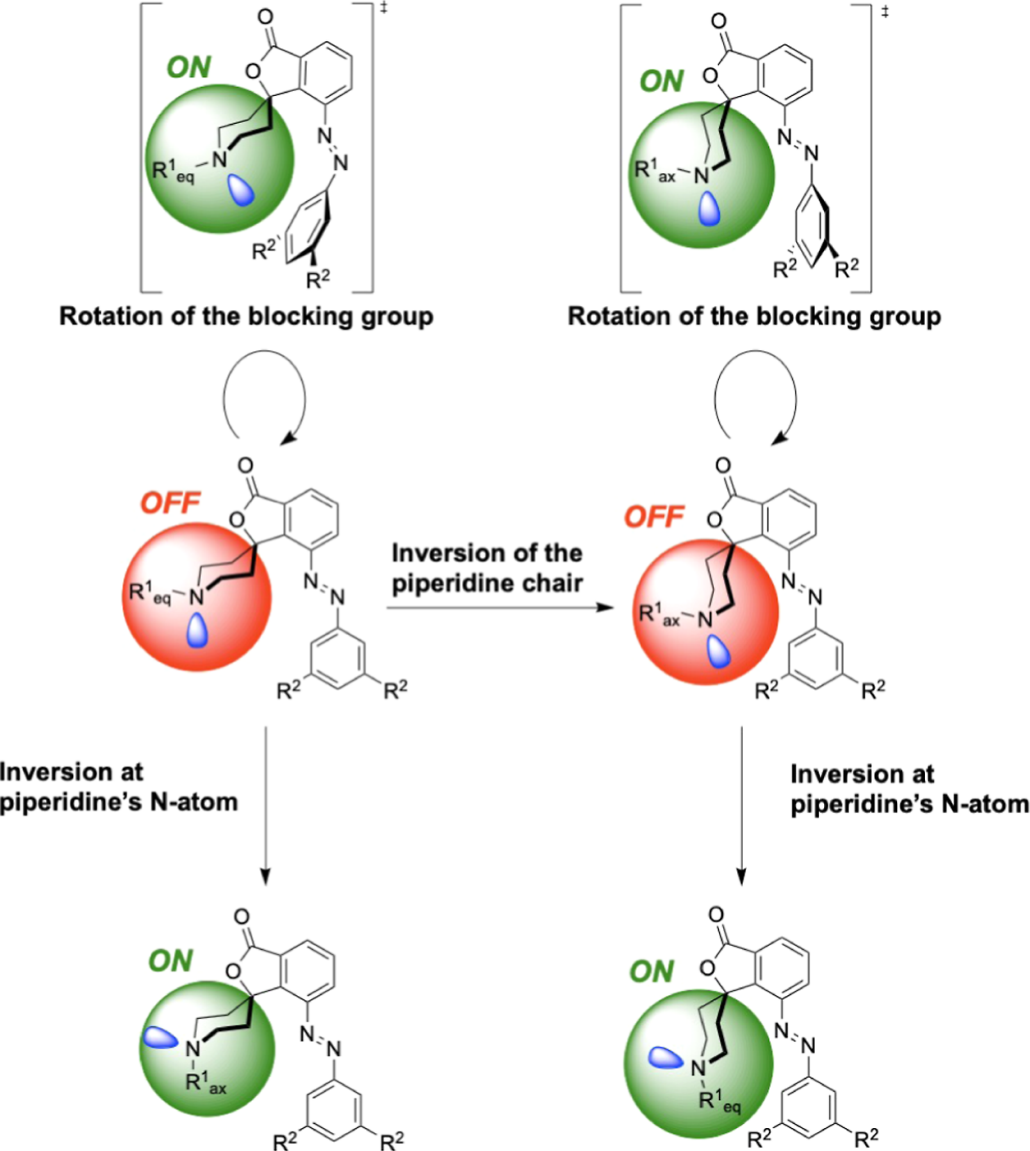
Representative Local Minima for (*E*)-PS1/2 Highlighting Routes to “False OFF” States

In the (*E*)-configuration, where the catalyst should be inactive, two processes are mainly responsible for the population of false OFF states (i.e., states that display catalytic activity when they are expected to be inactive): inversion of the piperidine N atom (Scheme 2, bottom) and rotation of the blocking group around the (N=N)–Ph bond that places the benzofuranone and phenylazo groups in an orthogonal orientation (Scheme 2, top). The first process is associated with the basins lying in region **II**, while the second is associated with larger conformational entropic contributions to basin **I** (shown as an increased basin size, Figure 6). Examining the differences between the FESs computed at the DFTB level (Figure 6, top) and those employing the LKR–OMP correction (Figure 6, bottom) reveals notable discrepancies. For both PS1 and PS2, the LKR–OMP correction shows a significant decrease in the predicted probability of N-inversion. While the basins in regions **I** and **II** of (*E*)–PS1 are predicted to be nearly isoergonic at the DFTB level (0.3 kcal/mol), DFTB + LKR predicts basin **I** to be more stable by 1.5 kcal/mol. Similarly, for (*E*)-PS2, DFTB + LKR predicts a much more stable basin lying in region **I** than DFTB (4.2 vs 1.5 kcal/mol). The fact that basin **II** of PS2 is more destabilized than that of PS1 aligns well with the larger size of its R^1^ substituent and its unlikeliness of adopting an axial position in the piperidine ring due to unfavorable 1,3-diaxial interactions. Using the LKR–OMP correction also decreases the size of basins, yet the free energy differences between the red and green areas of **I** are unaffected [(*E*)-PS1: 1.1 vs 1.0 kcal/mol; (*E*)-PS2: 0.2 vs 0.3 kcal/mol].

**Figure 7:**
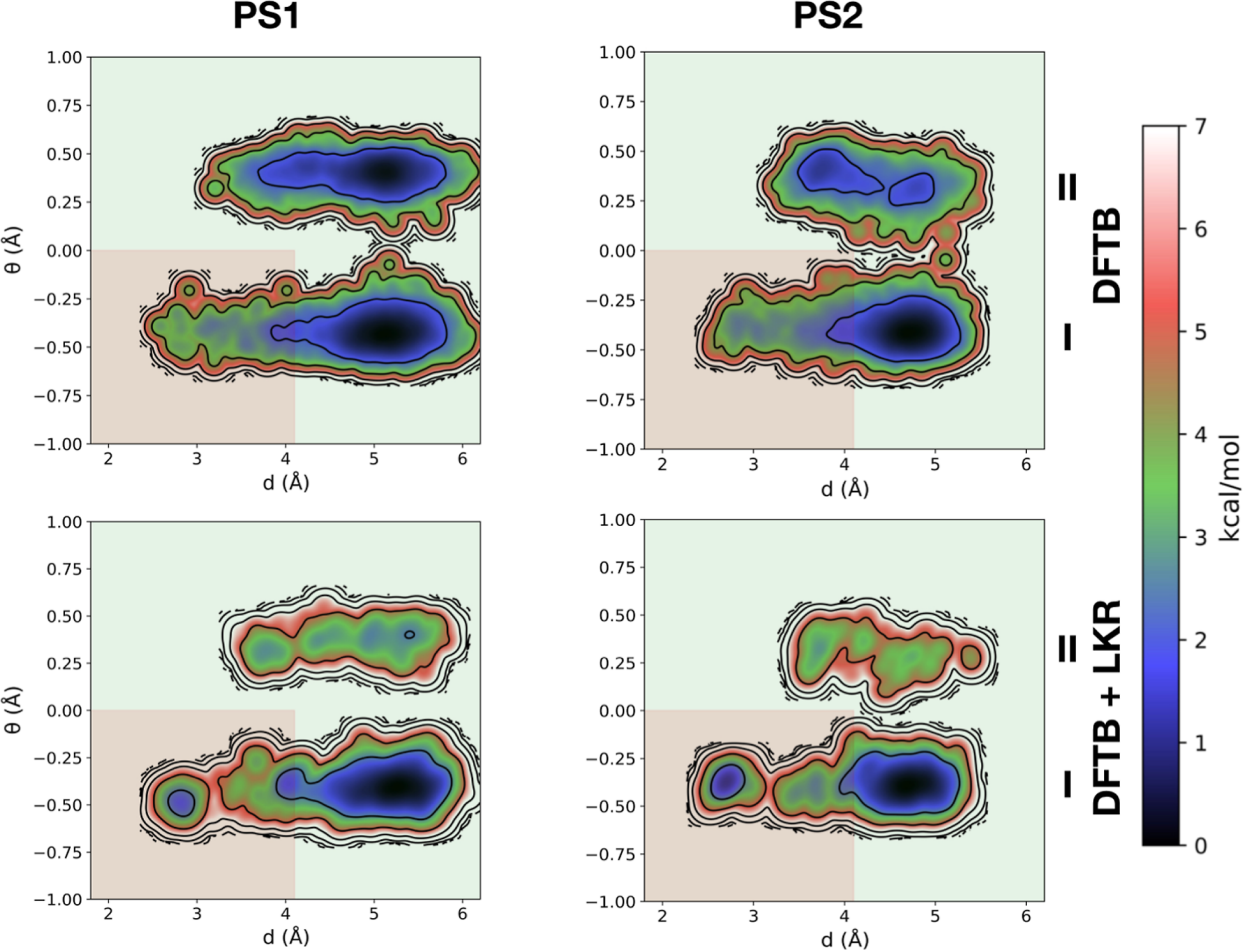
Computed FESs of (*Z*)-PS1/2 at the DFTB level with and without the LKR–OMP correction. The background colors correspond to the “OFF” [red, where the nitrogen atom lone pair (i.e., the active site) is sterically shielded] and “ON” (green, active site accessible to the substrate) states of the catalyst.

In the (*Z*)-configuration, the catalyst should be in its “ON” state, represented by basins lying in the green areas of Figure 7. As for the (*E*)-configuration, marked differences exist between the DFTB and the DFTB + LKR FESs. Notably, this includes the overstabilization of DFTB for basins lying in region **II** (corresponding to structures with an inverted axial R^1^ substituent), as well as a general broadening of the region **I** basin. While N-inversion does not affect catalytic activity in the (*Z*)-configuration, a broad basin in region **I** would correspond to undesired “false ON” states (i.e., structures lying in the red of region **I**). While DFTB predicts these regions to lie higher in energy, DFTB + LKR suggests that stable structures corresponding to these “false ON” states exist, particularly for PS2. The findings of DFTB + LKR indeed align well with reported experimental results: the (measured) yield of β-nitro alcohol is 72% for (*Z*)-PS1 but only 57% for (*Z*)-PS2.^104^ Essentially, PS2 has a less structured (*Z*)-state and can more easily access catalytically inactive conformations through rotation around the isobenzofuranone core. Conversely, (*Z*)-PS1 has a lower probability of visiting the red area of the FES thanks to the small size ratio of its R^1^ and R^2^ substituents and their inability to effectively shield the nitrogen’s lone pair in this configurational state. Overall, the limitation of the reversible steric shielding strategy, i.e., introducing a larger R^1^ substituent to minimize the population of false OFF states in the (*E*)-configuration leads to a more flexible (*Z*)-configuration where true ON states are less frequently visited, is evident.

Overall, the DFTB and DFTB + LKR FESs in both Figures 6 and 7 show notable differences, particularly with regard to the quantitative assessment of the thermodynamic stability of different states. Despite the existence of qualitative similarities between the two theoretical levels confirming that conformational states are highly flexible and driven by entropy and anharmonic effects, the presence of quantitative discrepancies would lead to a complete misidentification of problematic states. Specifically, analysis of only DFTB profiles would lead to “false OFF” states found in the (*E*)-configuration as being the most pressing concern, while analysis of the FES computed at the DFTB + LKR level shows that “false ON” states in the (*Z*)-configuration may actually be a more pressing issue for full catalytic control and an improved efficiency.

## 5 Conclusions

We introduced the OFF–ON database, a repository consisting of 7869 equilibrium and 67,457 out-of-equilibrium structures, representing the structural, chemical, and conformational diversities of any functionalized flexible organic molecule containing H, C, N, O, F, and S atoms. In particular, our effort focused on describing photoswitchable organocatalysts, challenging systems that require thorough exploration of their conformational and configurational space to fully grasp their catalytic behavior. Using the OFF–ON database, a LKR model (LKR–OMP) was trained to correct semiempirical energies (DFTB) to a hybrid DFT level (PBE0-D3). Combining this model with enhanced sampling techniques (e.g., resH–RE) provides access to the FES of virtually any (photoswitchable) organocatalyst, which allows the impact of different substituents on the conformational landscape of ON/OFF states to be probed and the resulting catalytic activity to be rationalized. Overall, the presented framework constitutes a general and fully transferable approach to extend the applicability of ML potentials to the sampling of complex molecular systems lacking obvious modularity. Crucially, this provides access to the chemical accuracy needed to depict the energetics of catalytic processes without sacrificing the exploration of the full FES. Comparisons of the DFTB level and the newly accessible PBE0-D3 level FESs for a set of prototypical photoswitchable organocatalysts reveal notable differences in anticipated behavior. Overall, this work should pave the way to the computationally led design of functional molecules by allowing rapid access to FESs of functional organic molecules with improved accuracy.

## Data Availability

The database curated in this work is openly available in the Materials Cloud at https://archive.materialscloud.org/record/2023.189. The LKR–OMP and resH–RE Python scripts are available on GitHub at https://github.com/lcmd-epfl.

## Supporting Information Available

Description of database curation with functional groups included and SMILES strings of structures, assessments of accuracy of the ML potential, analysis of local atomic contributions for the LKR–OMP correction, and convergence of the free energy profiles (PDF)

## References

[ref1] Gelžinytė E.; Öeren M.; Segall M. D.; Csányi G. Transferable Machine Learning Interatomic Potential for Bond Dissociation Energy Prediction of Drug-like Molecules. J. Chem. Theory Comput. 2024, 20, 164–177. 38108269 10.1021/acs.jctc.3c00710 PMC10782450

[ref2] Deringer V. L.; Caro M. A.; Csányi G. Machine Learning Interatomic Potentials as Emerging Tools for Materials Science. Adv. Mater. 2019, 31, 1902765 10.1002/adma.201902765. 31486179

[ref3] Vandenhaute S.; Cools-Ceuppens M.; DeKeyser S.; Verstraelen T.; Van Speybroeck V. Machine Learning Potentials for Metal-Organic Frameworks using an Incremental Learning Approach. npj Comput. Mater. 2023, 9, 19 10.1038/s41524-023-00969-x.

[ref4] Xu J.; Cao X.-M.; Hu P. Accelerating Metadynamics-Based Free-Energy Calculations with Adaptive Machine Learning Potentials. J. Chem. Theory Comput. 2021, 17, 4465–4476. 10.1021/acs.jctc.1c00261. 34100605

[ref5] Wieder M.; Fass J.; Chodera J. D. Fitting Quantum Machine Learning Potentials to Experimental Free Energy Data: Predicting Tautomer Ratios in Solution. Chem. Sci. 2021, 12, 11364–11381. 10.1039/D1SC01185E. 34567495 PMC8409483

[ref6] Dral P. O.; Owens A.; Dral A.; Csányi G. Hierarchical Machine Learning of Potential Energy Surfaces. J. Chem. Phys. 2020, 152, 204110 10.1063/5.0006498. 32486656

[ref7] Jaffrelot Inizan T.; Plé T.; Adjoua O.; Ren P.; Gökcan H.; Isayev O.; Lagardère L.; Piquemal J.-P. Scalable Hybrid Deep Neural Networks/Polarizable Potentials Biomolecular Simulations Including Long-Range Effects. Chem. Sci. 2023, 14, 5438–5452. 10.1039/d2sc04815a. 37234902 PMC10208042

[ref8] Liu W.; Zhu Y.; Wu Y.; Chen C.; Hong Y.; Yue Y.; Zhang J.; Hou B. Molecular Dynamics and Machine Learning in Catalysts. Catalysts 2021, 11, 1129 10.3390/catal11091129.

[ref9] Behler J. Perspective: Machine Learning Potentials for Atomistic Simulations. J. Chem. Phys. 2016, 145, 170901 10.1063/1.4966192. 27825224

[ref10] Behler J. First Principles Neural Network Potentials for Reactive Simulations of Large Molecular and Condensed Systems. Angew. Chem., Int. Ed. 2017, 56, 12828–12840. 10.1002/anie.201703114. 28520235

[ref11] Cheng B.; Engel E. A.; Behler J.; Dellago C.; Ceriotti M. Ab Initio Thermodynamics of Liquid and Solid Water. Proc. Natl. Acad. Sci. U.S.A. 2019, 116, 1110–1115. 10.1073/pnas.1815117116. 30610171 PMC6347673

[ref12] Morawietz T.; Singraber A.; Dellago C.; Behler J. How van der Waals Interactions Determine the Unique Properties of Water. Proc. Natl. Acad. Sci. U.S.A. 2016, 113, 8368–8373. 10.1073/pnas.1602375113. 27402761 PMC4968748

[ref13] Rossi K.; Jurásková V.; Wischert R.; Garel L.; Corminbœuf C.; Ceriotti M. Simulating Solvation and Acidity in Complex Mixtures with First-Principles Accuracy: The Case of CH_3_SO_3_H and H_2_O_2_ in Phenol. J. Chem. Theory Comput. 2020, 16, 5139–5149. 10.1021/acs.jctc.0c00362. 32567854

[ref14] Jurásková V.; Célerse F.; Laplaza R.; Corminboeuf C. Assessing the Persistence of Chalcogen Bonds in Solution with Neural Network Potentials. J. Chem. Phys. 2022, 156, 154112 10.1063/5.0085153. 35459295

[ref15] Gkeka P.; Stoltz G.; Barati Farimani A.; Belkacemi Z.; Ceriotti M.; Chodera J. D.; Dinner A. R.; Ferguson A. L.; Maillet J.-B.; Minoux H.; Peter C.; Pietrucci F.; Silveira A.; Tkatchenko A.; Trstanova Z.; Wiewiora R.; et al. Machine Learning Force Fields and Coarse-Grained Variables in Molecular Dynamics: Application to Materials and Biological Systems. J. Chem. Theory Comput. 2020, 16, 4757–4775. 10.1021/acs.jctc.0c00355. 32559068 PMC8312194

[ref16] Westermayr J.; Marquetand P. Machine Learning and Excited-State Molecular Dynamics. Mach. Learn.: Sci. Technol. 2020, 1, 043001 10.1088/2632-2153/ab9c3e.

[ref17] Wang J.; Olsson S.; Wehmeyer C.; Pérez A.; Charron N. E.; De Fabritiis G.; Noé F.; Clementi C. Machine Learning of Coarse-Grained Molecular Dynamics Force Fields. ACS Cent. Sci. 2019, 5, 755–767. 10.1021/acscentsci.8b00913. 31139712 PMC6535777

[ref18] Pattnaik P.; Raghunathan S.; Kalluri T.; Bhimalapuram P.; Jawahar C.; Priyakumar U. D. Machine Learning for Accurate Force Calculations in Molecular Dynamics Simulations. J. Phys. Chem. A 2020, 124, 6954–6967. 10.1021/acs.jpca.0c03926. 32786995

[ref19] Suwa H.; Smith J. S.; Lubbers N.; Batista C. D.; Chern G.-W.; Barros K. Machine Learning for Molecular Dynamics with Strongly Correlated Electrons. Phys. Rev. B 2019, 99, 161107 10.1103/PhysRevB.99.161107.

[ref20] Böselt L.; Thürlemann M.; Riniker S. Machine Learning in QM/MM Molecular Dynamics Simulations of Condensed-Phase Systems. J. Chem. Theory Comput. 2021, 17, 2641–2658. 10.1021/acs.jctc.0c01112. 33818085

[ref21] Hong S. J.; Chun H.; Lee J.; Kim B.-H.; Seo M. H.; Kang J.; Han B. First-Principles-Based Machine-Learning Molecular Dynamics for Crystalline Polymers with van der Waals Interactions. J. Phys. Chem. Lett. 2021, 12, 6000–6006. 10.1021/acs.jpclett.1c01140. 34165310

[ref22] Häse F.; Fdez Galván I.; Aspuru-Guzik A.; Lindh R.; Vacher M. How Machine Learning Can Assist the Interpretation of Ab Initio Molecular Dynamics Simulations and Conceptual Understanding of Chemistry. Chem. Sci. 2019, 10, 2298–2307. 10.1039/c8sc04516j. 30881655 PMC6385677

[ref23] Zeng J.; Cao L.; Xu M.; Zhu T.; Zhang J. Z. Complex Reaction Processes in Combustion Unraveled by Neural Network-Based Molecular Dynamics Simulation. Nat. Commun. 2020, 11, 5713 10.1038/s41467-020-19497-z. 33177517 PMC7658983

[ref24] Chmiela S.; Tkatchenko A.; Sauceda H. E.; Poltavsky I.; Schütt K. T.; Müller K. R. Machine Learning of Accurate Energy-Conserving Molecular Force Fields. Sci. Adv. 2017, 3, e1603015 10.1126/sciadv.1603015. 28508076 PMC5419702

[ref25] Behler J.; Parrinello M. Generalized Neural-Network Representation of High-Dimensional Potential-Energy Surfaces. Phys. Rev. Lett. 2007, 98, 146401 10.1103/PhysRevLett.98.146401. 17501293

[ref26] Smith J. S.; Isayev O.; Roitberg A. E. ANI-1: An Extensible Neural Network Potential with DFT Accuracy at Force Field Computational Cost. Chem. Sci. 2017, 8, 3192–3203. 10.1039/C6SC05720A. 28507695 PMC5414547

[ref27] Wang H.; Zhang L.; Han J.; Weinan E. DeePMD-Kit: A Deep Learning Package for Many-Body Potential Energy Representation and Molecular Dynamics. Comput. Phys. Commun. 2018, 228, 178–184. 10.1016/j.cpc.2018.03.016.

[ref28] Schütt K. T.; Sauceda H. E.; Kindermans P.-J.; Tkatchenko A.; Müller K.-R. SchNet – A deep learning architecture for molecules and materials. J. Chem. Phys. 2018, 148, 241722 10.1063/1.5019779. 29960322

[ref29] Unke O. T.; Meuwly M. PhysNet A Neural Network for Predicting Energies, Forces, Dipole Moments, and Partial Charges. J. Chem. Theory Comput. 2019, 15, 3678–3693. 10.1021/acs.jctc.9b00181. 31042390

[ref30] Unke O. T.; Chmiela S.; Gastegger M.; Schütt K. T.; Sauceda H. E.; Müller K. R. SpookyNet: Learning Force Fields With Electronic Degrees of Freedom and Nonlocal Effects. Nat. Commun. 2021, 12, 7273 10.1038/s41467-021-27504-0. 34907176 PMC8671403

[ref31] Batzner S.; Musaelian A.; Sun L.; Geiger M.; Mailoa J. P.; Kornbluth M.; Molinari N.; Smidt T. E.; Kozinsky B. E(3)- Graph Neural Networks for Data-Efficient and Accurate Interatomic Potentials. Nat. Commun. 2022, 13, 2453 10.1038/s41467-022-29939-5. 35508450 PMC9068614

[ref32] Bartók A. P.; Payne M. C.; Kondor R.; Csányi G. Gaussian Approximation Potentials: The Accuracy of Quantum Mechanics, Without the Electrons. Phys. Rev. Lett. 2010, 104, 136403 10.1103/PhysRevLett.104.136403. 20481899

[ref33] Bartók-Pártay A. The Gaussian Approximation Potential: An Interatomic Potential Derived from First Principles Quantum Mechanics; Springer Science & Business Media, 2010.

[ref34] John S.; Csányi G. Many-Body Coarse-Grained Interactions Using Gaussian Approximation Potentials. J. Phys. Chem. B 2017, 121, 10934–10949. 10.1021/acs.jpcb.7b09636. 29117675

[ref35] George J.; Hautier G.; Bartók A. P.; Csányi G.; Deringer V. L. Combining Phonon Accuracy With High Transferability in Gaussian Approximation Potential Models. J. Chem. Phys. 2020, 153, 044104 10.1063/5.0013826. 32752705

[ref36] Vovk V. Empirical Inference; Springer, 2013; pp 105–116.

[ref37] Deringer V. L.; Bartók A. P.; Bernstein N.; Wilkins D. M.; Ceriotti M.; Csányi G. Gaussian Process Regression for Materials and Molecules. Chem. Rev. 2021, 121, 10073–10141. 10.1021/acs.chemrev.1c00022. 34398616 PMC8391963

[ref38] Balabin R. M.; Lomakina E. I. Support Vector Machine Regression (LS-SVM)—An Alternative to Artificial Neural Networks (ANNs) for the Analysis of Quantum Chemistry Data?. Phys. Chem. Chem. Phys. 2011, 13, 11710–11718. 10.1039/c1cp00051a. 21594265

[ref39] Ho T.-S.; Rabitz H. A General Method for Constructing Multidimensional Molecular Potential Energy Surfaces from Ab Initio Calculations. J. Phys. Chem. 1996, 104, 2584–2597. 10.1063/1.470984. 15012421

[ref40] Unke O. T.; Meuwly M. Toolkit for the Construction of Reproducing Kernel-Based Representations of Data: Application to Multidimensional Potential Energy Surfaces. J. Chem. Inf. Model. 2017, 57, 1923–1931. 10.1021/acs.jcim.7b00090. 28666387

[ref41] Chmiela S.; Sauceda H. E.; Müller K. R.; Tkatchenko A. Towards Exact Molecular Dynamics Simulations with Machine-Learned Force Fields. Nat. Commun. 2018, 9, 3887 10.1038/s41467-018-06169-2. 30250077 PMC6155327

[ref42] Sauceda H. E.; Chmiela S.; Poltavsky I.; Müller K. R.; Tkatchenko A. Molecular Force Fields With Gradient-Domain Machine Learning: Construction and Application to Dynamics of Small Molecules with Coupled Cluster Forces. J. Chem. Phys. 2019, 150, 114102 10.1063/1.5078687. 30901990

[ref43] Ruddigkeit L.; Van Deursen R.; Blum L. C.; Reymond J.-L. Enumeration of 166 Billion Organic Small Molecules in the Chemical Universe Database GDB-17. J. Chem. Inf. Model. 2012, 52, 2864–2875. 10.1021/ci300415d. 23088335

[ref44] Gaulton A.; Bellis L. J.; Bento A. P.; Chambers J.; Davies M.; Hersey A.; Light Y.; McGlinchey S.; Michalovich D.; Al-Lazikani B.; Overington J. P. ChEMBL: A Large-Scale Bioactivity Database for Drug Discovery. Nucleic Acids Res. 2012, 40, D1100–D1107. 10.1093/nar/gkr777. 21948594 PMC3245175

[ref45] Allen F. H. The Cambridge Structural Database: A Quarter of a Million Crystal Structures and Rising. Acta Crystallogr., Sect. B: Struct. Sci. 2002, 58, 380–388. 10.1107/S0108768102003890. 12037359

[ref46] Sterling T.; Irwin J. J. ZINC 15–Ligand Discovery for Everyone. J. Chem. Inf. Model. 2015, 55, 2324–2337. 10.1021/acs.jcim.5b00559. 26479676 PMC4658288

[ref47] Kim S.; Chen J.; Cheng T.; Gindulyte A.; He J.; He S.; Li Q.; Shoemaker B. A.; Thiessen P. A.; Yu B.; Zaslavsky L.; Zhang J.; Bolton E. E. PubChem 2019 Update: Improved Access to Chemical Data. Nucleic Acids Res. Spec. Publ. 2019, 47, D1102–D1109. 10.1093/nar/gky1033. PMC6324075 30371825

[ref48] Sorokina M.; Merseburger P.; Rajan K.; Yirik M. A.; Steinbeck C. COCONUT Online: Collection of Open Natural Products Database. J. Cheminform. 2021, 13, 2 10.1186/s13321-020-00478-9. 33423696 PMC7798278

[ref49] Wishart D. S.; Knox C.; Guo A. C.; Cheng D.; Shrivastava S.; Tzur D.; Gautam B.; Hassanali M. DrugBank: A Knowledgebase for Drugs, Drug Actions and Drug Targets. Nucleic Acids Res. 2008, 36, D901–D906. 10.1093/nar/gkm958. 18048412 PMC2238889

[ref50] Martins P. M.; Santos L. H.; Mariano D.; Queiroz F. C.; Bastos L. L.; Gomes I. d. S.; Fischer P. H.; Rocha R. E.; Silveira S. A.; de Lima H.; Mariana T.; Maria G.; Raquel C. Propedia: A Database for Protein–Peptide Identification Based on a Hybrid Clustering Algorithm. BMC Bioinf. 2021, 22, 1 10.1186/s12859-020-03881-z. PMC7776311 33388027

[ref51] Shtatland T.; Guettler D.; Kossodo M.; Pivovarov M.; Weissleder R. PepBank-A Database of Peptides Based on Sequence Text Mining and Public Peptide Data Sources. BMC Bioinf. 2007, 8, 280 10.1186/1471-2105-8-280. PMC1976427 17678535

[ref52] Das D.; Jaiswal M.; Khan F. N.; Ahamad S.; Kumar S. PlantPepDB: A Manually Curated Plant Peptide Database. Sci. Rep. 2020, 10, 2194 10.1038/s41598-020-59165-2. 32042035 PMC7010657

[ref53] Christensen A. S.; Sirumalla S. K.; Qiao Z.; O’Connor M. B.; Smith D. G.; Ding F.; Bygrave P. J.; Anandkumar A.; Welborn M.; Manby F. R.; Miller T. F. OrbNet Denali: A Machine Learning Potential for Biological and Organic Chemistry with Semi-Empirical Cost and DFT Accuracy. J. Chem. Phys. 2021, 155, 204103 10.1063/5.0061990. 34852495

[ref54] Pinheiro M. Jr.; Zhang S.; Dral P. O.; Barbatti M. WS22 Database, Wigner Sampling and Geometry Interpolation for Configurationally Diverse Molecular Datasets. Sci. Data 2023, 10, 95 10.1038/s41597-023-01998-3. 36792601 PMC9931705

[ref55] Eastman P.; Behara P. K.; Dotson D. L.; Galvelis R.; Herr J. E.; Horton J. T.; Mao Y.; Chodera J. D.; Pritchard B. P.; Wang Y.; De Fabritiis G.; Markland T. E. SPICE, A Dataset of Drug-Like Molecules and Peptides for Training Machine Learning Potentials. Sci. Data 2023, 10, 11 10.1038/s41597-022-01882-6. 36599873 PMC9813265

[ref56] Schmitz G.; Godtliebsen I. H.; Christiansen O. Machine Learning for Potential Energy Surfaces: An Extensive Database and Assessment of Methods. J. Chem. Phys. 2019, 150, 244113 10.1063/1.5100141. 31255074

[ref57] Schreiner M.; Bhowmik A.; Vegge T.; Busk J.; Winther O. Transition1x - A Dataset for Building Generalizable Reactive Machine Learning Potentials. Sci. Data 2022, 9, 779 10.1038/s41597-022-01870-w. 36566281 PMC9789978

[ref58] Nguyen M.-T.; Rousseau R.; Paviet P. D.; Glezakou V.-A. Actinide Molten Salts: A Machine-Learning Potential Molecular Dynamics Study. ACS Appl. Mater. Interfaces 2021, 13, 53398–53408. 10.1021/acsami.1c11358. 34494435

[ref59] Grill K.; Dube H. Supramolecular Relay-Control of Organocatalysis with a Hemithioindigo-Based Molecular Motor. J. Am. Chem. Soc. 2020, 142, 19300–19307. 10.1021/jacs.0c09519. 33112151

[ref60] Sheng J.; Pooler D. R.; Feringa B. L. Enlightening Dynamic Functions in Molecular Systems by Intrinsically Chiral Light-Driven Molecular Motors. Chem. Soc. Rev. 2023, 52, 5875–5891. 10.1039/D3CS00247K. 37581608 PMC10464662

[ref61] Ihrig S. P.; Eisenreich F.; Hecht S. Photoswitchable Polymerization Catalysis: State of the Art, Challenges, and Perspectives. Chem. Commun. 2019, 55, 4290–4298. 10.1039/C9CC01431D. 30924476

[ref62] Dorel R.; Feringa B. L. Photoswitchable Catalysis based on the Isomerisation of Double Bonds. Chem. Commun. 2019, 55, 6477–6486. 10.1039/C9CC01891C. 31099809

[ref63] Li J.; Hecht S. Versatile Photoswitchable Molecules in Catalysis; Wiley Online Library, 2022.

[ref64] Majee D.; Presolski S. Dithienylethene-Based Photoswitchable Catalysts: State of the Art and Future Perspectives. ACS Catal. 2021, 11, 2244–2252. 10.1021/acscatal.0c05232.

[ref65] Liu R.; Zhang X.; Xia F.; Dai Y. Azobenzene-Based Photoswitchable Catalysts: State of the Art and Perspectives. J. Catal. 2022, 409, 33–40. 10.1016/j.jcat.2022.03.015.

[ref66] Osorio-Planes L.; Rodriguez-Escrich C.; Pericàs M. A. Photoswitchable Thioureas for the External Manipulation of Catalytic Activity. Org. Lett. 2014, 16, 1704–1707. 10.1021/ol500381c. 24611899

[ref67] Freixa Z. Photoswitchable Catalysis Using Organometallic Complexes. Catal. Sci. Technol. 2020, 10, 3122–3139. 10.1039/D0CY00431F.

[ref68] Imahori T.; Yamaguchi R.; Kurihara S. Azobenzene-Tethered Bis (Trityl Alcohol) as a Photoswitchable Cooperative Acid Catalyst for Morita–Baylis–Hillman Reactions. Chem. —Eur. J. 2012, 18, 10802–10807. 10.1002/chem.201201383. 22806895

[ref69] Pizzolato S. F.; Collins B. S.; van Leeuwen T.; Feringa B. L. Bifunctional Molecular Photoswitches Based on Overcrowded Alkenes for Dynamic Control of Catalytic Activity in Michael Addition Reactions. Chem. —Eur. J. 2017, 23, 6174–6184. 10.1002/chem.201604966. 27880015

[ref70] De Bo G.; Leigh D. A.; McTernan C. T.; Wang S. A Complementary Pair of Enantioselective Switchable Organocatalysts. Chem. Sci. 2017, 8, 7077–7081. 10.1039/C7SC02462B. 29147536 PMC5637462

[ref71] Vlatković M.; Bernardi L.; Otten E.; Feringa B. L. Dual Stereocontrol Over the Henry Reaction Using a Light–And Heat-Triggered Organocatalyst. Chem. Commun. 2014, 50, 7773–7775. 10.1039/c4cc00794h. 24667967

[ref72] Griffiths R.-R.; Greenfield J. L.; Thawani A. R.; Jamasb A. R.; Moss H. B.; Bourached A.; Jones P.; McCorkindale W.; Aldrick A. A.; Fuchter M. J.; Lee A. A. Data-Driven Discovery of Molecular Photoswitches with Multioutput Gaussian Processes. Chem. Sci. 2022, 13, 13541–13551. 10.1039/D2SC04306H. 36507171 PMC9682911

[ref73] Vela S.; Krüger C.; Corminboeuf C. Exploring Chemical Space in the Search for Improved Azoheteroarene-Based Photoswitches. Phys. Chem. Chem. Phys. 2019, 21, 20782–20790. 10.1039/C9CP03831K. 31513205

[ref74] Vela S.; Corminboeuf C. The Photoisomerization Pathway(s) of Push–Pull Phenylazoheteroarenes. Chem. —Eur. J. 2020, 26, 14724–14729. 10.1002/chem.202002321. 32692427 PMC7756763

[ref75] Vela S.; Scheidegger A.; Fabregat R.; Corminboeuf C. Tuning the Thermal Stability and Photoisomerization of Azoheteroarenes through Macrocycle Strain. Chem. —Eur. J. 2021, 27, 419–426. 10.1002/chem.202003926. 32991023 PMC7839710

[ref76] Gallarati S.; Fabregat R.; Juraskova V.; Inizan T. J.; Corminboeuf C. How Robust Is the Reversible Steric Shielding Strategy for Photoswitchable Organocatalysts?. J. Org. Chem. 2022, 87, 8849–8857. 10.1021/acs.joc.1c02991. 35762705 PMC9295146

[ref77] Gallarati S.; van Gerwen P.; Laplaza R.; Vela S.; Fabrizio A.; Corminboeuf C. OSCAR: An Extensive Repository of Chemically and Functionally Diverse Organocatalysts. Chem. Sci. 2022, 13, 13782–13794. 10.1039/D2SC04251G. 36544722 PMC9710326

[ref78] Gallarati S.; Laplaza R.; Corminboeuf C. Harvesting the Fragment-Based Nature of Bifunctional Organocatalysts to Enhance Their Activity. Org. Chem. Front. 2022, 9, 4041–4051. 10.1039/D2QO00550F.

[ref79] Fabregat R.; Fabrizio A.; Engel E. A.; Meyer B.; Juraskova V.; Ceriotti M.; Corminboeuf C. Local Kernel Regression and Neural Network Approaches to the Conformational Landscapes of Oligopeptides. J. Chem. Theory Comput. 2022, 18, 1467–1479. 10.1021/acs.jctc.1c00813. 35179897 PMC8908737

[ref80] Ramakrishnan R.; Dral P. O.; Rupp M.; von Lilienfeld O. A. Big Data Meets Quantum Chemistry Approximations: The Δ-Machine Learning Approach. J. Chem. Theory Comput. 2015, 11, 2087–2096. 10.1021/acs.jctc.5b00099. 26574412

[ref81] Groom C. R.; Bruno I. J.; Lightfoot M. P.; Ward S. C. The Cambridge Structural Database. Acta Crystallogr., Sect. B: Struct. Sci., Cryst. Eng. Mater. 2016, 72, 171–179. 10.1107/S2052520616003954. PMC4822653 27048719

[ref82] Řezáč J. Non-Covalent Interactions Atlas Benchmark Data Sets: Hydrogen Bonding. J. Chem. Theory Comput. 2020, 16, 2355–2368. 10.1021/acs.jctc.9b01265. 32149503

[ref83] Řezáč J. Non-Covalent Interactions Atlas Benchmark Data Sets 5: London Dispersion in an Extended Chemical Space. Phys. Chem. Chem. Phys. 2022, 24, 14780–14793. 10.1039/D2CP01602H. 35686612

[ref84] Řezáč J. Non-Covalent Interactions Atlas Benchmark Data Sets 2: Hydrogen Bonding in an Extended Chemical Space. J. Chem. Theory Comput. 2020, 16, 6305–6316. 10.1021/acs.jctc.0c00715. 32941026

[ref85] Kubillus M.; Kubař T.; Gaus M.; Řezáč J.; Elstner M. Parameterization of the DFTB3 Method for Br, Ca, Cl, F, I, K, and Na in Organic and Biological Systems. J. Chem. Theory Comput. 2015, 11, 332–342. 10.1021/ct5009137. 26889515

[ref86] Gaus M.; Lu X.; Elstner M.; Cui Q. Parameterization of DFTB3/3OB for Sulfur and Phosphorus for Chemical and Biological Applications. J. Chem. Theory Comput. 2014, 10, 1518–1537. 10.1021/ct401002w. 24803865 PMC3985940

[ref87] Hourahine B.; Aradi B.; Blum V.; Bonafé F.; Buccheri A.; Camacho C.; Cevallos C.; Deshaye M.; Dumitrică T.; Dominguez A.; Ehlert S.; Elstner M.; van der Heide T.; Hermann J.; Irle S.; Kranz J. J.; Kohler C.; Kowalczyk T.; Kubař T.; Lee I. S.; Lutsker V.; Maurer R. J.; Min S. K.; Mitchell I.; Negre C.; Niehaus T. A.; Niklasson A. M. N.; Page A. J.; Pecchia A.; Penazzi G.; Persson M. P.; Rezac J.; Sánchez C. G.; Sternberg M.; Stohr M.; Stuckenberg F.; Tkatchenko A.; Yu V. W.; Frauenheim T. DFTB+, A Software Package for Efficient Approximate Density Functional Theory Based Atomistic Simulations. J. Chem. Phys. 2020, 152, 124101 10.1063/1.5143190. 32241125

[ref88] Perdew J. P.; Burke K.; Ernzerhof M. Generalized Gradient Approximation Made Simple. Phys. Rev. Lett. 1996, 77, 3865–3868. 10.1103/PhysRevLett.77.3865. 10062328

[ref89] Adamo C.; Barone V. Toward Reliable Density Functional Methods without Adjustable Parameters: The PBE0 Model. J. Chem. Phys. 1999, 110, 6158–6170. 10.1063/1.478522.

[ref90] Moellmann J.; Grimme S. DFT-D3 Study of Some Molecular Crystals. J. Phys. Chem. C 2014, 118, 7615–7621. 10.1021/jp501237c.

[ref91] Weigend F.; Ahlrichs R. Balanced Basis Sets of Split Valence, Triple Zeta Valence and Quadruple Zeta Valence Quality for H to Rn: Design and Assessment of Accuracy. Phys. Chem. Chem. Phys. 2005, 7, 3297–3305. 10.1039/b508541a. 16240044

[ref92] Seritan S.; Bannwarth C.; Fales B. S.; Hohenstein E. G.; Kokkila-Schumacher S. I.; Luehr N.; Snyder J. W.; Song C.; Titov A. V.; Ufimtsev I. S.; Martinez T. J. TeraChem: Accelerating Electronic Structure and Ab Initio Molecular Dynamics With Graphical Processing Units. J. Chem. Phys. 2020, 152, 224110 10.1063/5.0007615. 32534542 PMC7928072

[ref93] Christensen A. S.; Bratholm L. A.; Faber F. A.; von Lilienfeld O. A. FCHL Revisited: Faster and More Accurate Quantum Machine Learning. J. Chem. Phys. 2020, 152, 044107 10.1063/1.5126701. 32007071

[ref94] Brandenburg J. G.; Grimme S. Accurate Modeling of Organic Molecular Crystals by Dispersion-Corrected Density Functional Tight Binding (DFTB). J. Phys. Chem. Lett. 2014, 5, 1785–1789. 10.1021/jz500755u. 26273854

[ref95] Tropp J. A.; Gilbert A. C. Signal Recovery From Random Measurements via Orthogonal Matching Pursuit. IEEE Trans. Inf. Theory 2007, 53, 4655–4666. 10.1109/TIT.2007.909108.

[ref96] Imbalzano G.; Anelli A.; Giofré D.; Klees S.; Behler J.; Ceriotti M. Automatic Selection of Atomic Fingerprints and Reference Configurations for Machine-Learning Potentials. J. Chem. Phys. 2018, 148, 241730 10.1063/1.5024611. 29960368

[ref97] Christensen A.; Faber F.; Huang B.; Bratholm L.; Tkatchenko A.; Muller K.; von Lilienfeld O. A. QML: A Python Toolkit for Quantum Machine Learning, 2017. https://github.com/qmlcode/qml.

[ref98] Petraglia R.; Nicolaï A.; Wodrich M. D.; Ceriotti M.; Corminboeuf C. Beyond Static Structures: Putting Forth REMD as a Tool to Solve Problems in Computational Organic Chemistry. J. Comput. Chem. 2016, 37, 83–92. 10.1002/jcc.24025. 26228927 PMC5324590

[ref99] Bussi G.; Parrinello M. Accurate Sampling Using Langevin Dynamics. Phys. Rev. E: Stat., Nonlinear, Soft Matter Phys. 2007, 75, 056707 10.1103/PhysRevE.75.056707. 17677198

[ref100] Huang B.; von Lilienfeld O. A. Quantum Machine Learning Using Atom-in-Molecule-Based Fragments Selected on the Fly. Nat. Chem. 2020, 12, 945–951. 10.1038/s41557-020-0527-z. 32929248

[ref101] Fabregat R.; Fabrizio A.; Meyer B.; Hollas D.; Corminboeuf C. Hamiltonian-Reservoir Replica Exchange and Machine Learning Potentials for Computational Organic Chemistry. J. Chem. Theory Comput. 2020, 16, 3084–3094. 10.1021/acs.jctc.0c00100. 32212720 PMC7704029

[ref102] Peters M. V.; Stoll R. S.; Kühn A.; Hecht S. Photoswitching of Basicity. Angew. Chem., Int. Ed. 2008, 47, 5968–5972. 10.1002/anie.200802050. 18624316

[ref103] Iida H.; Umebayashi N.; Yashima E. Photoswitchable Organocatalysis in Acylation of Alcohol Using Dithienylethene-Linked Azoles. Tetrahedron 2013, 69, 11064–11069. 10.1016/j.tet.2013.11.015.

[ref104] Stoll R. S.; Peters M. V.; Kuhn A.; Heiles S.; Goddard R.; Bühl M.; Thiele C. M.; Hecht S. Photoswitchable Catalysts: Correlating Structure and Conformational Dynamics with Reactivity by a Combined Experimental and Computational Approach. J. Am. Chem. Soc. 2009, 131, 357–367. 10.1021/ja807694s. 19061327

