# Supplementary material for: From Organic Fragments to Photoswitchable Catalysts: The OFF–ON Structural Repository for Transferable Kernel-Based Potentials
Source: J Chem Inf Model. 2024 Feb 6;64(4):1201–12. doi: 10.1021/acs.jcim.3c01953 (PMC10900300; doi:10.1021/acs.jcim.3c01953)
Supplement: Supplementary file 1 — ci3c01953_si_001.pdf [file ci3c01953_si_001.pdf]

Supporting Information
From Organic Fragments to Photoswitchable Catalysts: The OFF-ON Structural Repository for Transferable Kernel-based Potentials

Frédéric Célerse,† Matthew D. Wodrich,†,‡ Sergi Vela,† Simone Gallarati,† Raimon Fabregat,† Veronika Juraskova,†,‡ and Clémence Corminboeuf*,†,¶,‡

†*Laboratory for Computational Molecular Design (LCMD), Institute of Chemical Sciences and Engineering, Ecole Polytechnique Fédérale de Lausanne (EPFL), Lausanne, 1015, Switzerland.*

‡*National Center for Competence in Research-Catalysis (NCCR-Catalysis), École Polytechnique Fédérale de Lausanne, 1015 Lausanne, Switzerland*

¶*National Centre for Computational Design and Discovery of Novel Materials (MARVEL), École Polytechnique Fédérale de Lausanne, 1015 Lausanne, Switzerland*

E-mail: clemence.corminboeuf@epfl.ch

1 Database curation: protocol

1.1 A: Collect fragments and organic molecules

First, an initial pool of molecules was generated, including 1000 species from the OSCAR "seed" dataset,[1] 23 photoswitchable organocatalysts from the literature,[2–16] and 344 experimentally characterized azobenzene derivatives from Ref. [17], with chemical complexity limited to six elements (H, C, N, O, F, and S). Atom–in–molecule (AMON)–based fragments up to seven heavy atoms[18] are generated from this pool, yielding 10,270 unique fragments. Akin to the strategy followed in building OSCAR,[1] *cell2mol*[19] was used to increase the chemical diversity by extracting an additional 24065 molecules (neutral, with fewer than 20 heavy atoms) from the Cambridge Structural Database.[20]

Proceeding in the above manner insures that the preliminary database of 34,335 compounds is chemically diverse, however, it is also too large to directly train a LKR model. To prune the database without sacrificing chemical diversity (*i.e.*, eliminating species with over-represented environments) all structures were classified based on the presence of 28 predefined functional groups (FGs, see Table S1) made from six elements (H,C,N,O,F,S). This is accomplished by determining the presence of FGs using substructural searches in RDKit[21] performed on molecular SMILES strings generated from xyz2mol.[22] A vector of 28 entries (v) was generated for each molecule, with each entry (v_i) being the number of occurrences of each FG. To do so, SMARTS representations of all FG were built, and a sub-structural search was performed in the SMILES of the 3,535 structures using RDKIT. The resulting vectors were then used to select the list of molecules that brings more chemical diversity if added to the database. Typically, those would be the molecules with the largest number of occurrences of any FG. However, a balance must be achieved between FG. Thus, all FG were associated with a target value ranging from 0 to 1000, depending on its occurrence in the entire dataset. A target vector (t) with target values for the 28 FG (t_i) was created. Then, the algorithm proceeded by choosing a list of structures whose sum of v minimizes the distance

with t. The best combination of molecules rendered a distance vector ($\vec{d} = \vec{t} - sum(v)$) with most entries being below 10, indicating that the target values were achieved without significant deviation. The only exception were aldehydes, thiols, and secondary and tertiary amines, which are scarce in the initial database composed of 10,270 AMON fragments and 24,065 prefiltered CSD molecules.

Table S1: Functional groups (FG) considered during the initial database curation.

Atom type	FG	Description
C	CH_3–CH_3	Alkyl
N	C(=O)N	Amide
N	R_2C=CNR_2	Enamine
N	NH_2R	1ary amine
N	NHR_2	2ary amine
N	NR_3	3ary amine
N	N=N	Azo
N	N–N	Hydrazine
N	C(=N)NH_2	Hydrazone
N	R_2C=$NR_2{}^+$	Iminium
N	RC#N	Cyano
N	NO_2	Nitro
O	ROH	Alcohol
O	R_2C=O	Ketone
O	RHC=O	Aldehyde
O	RCOOR	Ester
O	RO–OR	Peroxide
O	C-O–R	Ether
O	RCOOH	Carboxylic acid
F	C-F	Fluorine
S	RSH	Thiol
S	RCOSR	Carbo-thioester
S	C–CS–C	Thiocarbonyl
S	CSN	Thioamide
S	RCOSR	Thio-ether
Ring		6–mem ring
Ring		6–mem hetero ring
Ring		5–mem hetero ring

Following classification, the goal is to trim the dataset such that the best representation of each of the FGs can be obtained using the fewest number of compounds. Overall, completing this process leave a total of 3,533 compounds structures, as depicted in Fig. 1 in the main

text. Each structure were finally optimized at the DFTB–D3/3ob level of theory.

1.2 B: Add photochromic units

We then augmented the database with a set of molecules sourced from various photoswitches. In addition to the previously mentioned 344 photoswitches, additional species identified in the PubChem database,[23] corresponding to known molecules that have already been experimentally synthesized, were also extracted (see Table S2 for SMILES format). To limit the size of each molecule while preserving the photochromic unit, we employed the Murcko Scaffold method implemented in RDKit, which efficiently and automatically extracts the aromatic structures along with their linkers. Doing so allowed us to isolate the photochromic part of the molecule while limiting the size of each generated molecule. We applied this method to all the SMILES. After filtering duplicates and ensuring (especially for structures capable of adopting both E and Z conformations) that both forms were well represented, we obtained a total of 544 molecules that we optimized at the DFTB-D3/3ob level.

Table S2: List of photoswitch SMILES extracted from the PubChem repository[23]

**Diarylethene**	**SMILES**
1	c1(c(ccs1)[C]1[C](c2c(scc2)C)CCC1)C
2	c1(c(cco1)[C]1[C](c2c(occ2)C)CCC1)C
3	c1(c(ccs1)[C]1[C](c2c(occ2)C)CCC1)C
4	c1(c(csc1)[C]1[C](c2c(scc2)C)CCC1)C
5	c1(c(ccs1)[C]1[C](c2c(scc2)C(F)(F)(F))CC(F)(F)C1)C(F)(F)(F)
6	c1(c(ccs1)[C]1[C](c2c(scc2)C)C(F)(F)CC1(F)(F))C
7	c1(c(ccs1)[C]1[C](c2c(scc2)C)C(F)(F)C(F)(F)C1(F)(F))C
8	s1c(c(cc1)[C]1C(=O)OC(=O)[C]1c1c(scc1)C)C
9	s1c(c(cc1)/C=C\ c1c(scc1)C)C
10	s1c(c(cc1)/C=C\ c1c(occ1)C)C
11	o1c(c(cc1)/C=C\ c1c(occ1)C)C
12	s1c(c(nc1)[C]1[C](c2c(scc2)C)CCC1)C
13	s1c(c(nc1)[C]1C(F)(F)C(C([C]1c1c(scc1)C)(F)F)(F)F)C
14	s1c(c(nc1)[C]1[C](c2ncsc2C)CCC1)C
15	n1(c(c(C)sc1)[C]1[C](c2c(scn2)C)C(F)(F)C(F)(F)C1(F)(F))

Continued on next page

Table S2: List of photoswitch SMILES extracted from the PubChem repository[23] (Continued)

16	c1cccc2c1sc(C)c2[C]1[C](c2c(scc2)C)CCC1
17	s1c2c(c(c1C)[C]1[C](c3c(scc3)C)CCC1)cncc2
18	c1c(=O)oc(=O)c2c1sc(C)c2[C]1[C](c2c(scc2)C)CCC1
**Spiropyran and Merco-cyanine**	**SMILES**
1	C1CSC2(N1)C=CC=CO2
2	C1C2=CC=CC=C2N(C13C=CC4=C(O3)C=CC(=C4)[N+](=O)[O-])CCC(=O)O
3	C1=CC=C2C(=C1)C3(C=CC=CO3)SS2
4	C1=CC2=CC3(C=CC=CO3)C(=O)N=C2C=C1
5	C1=CC=C2C(=C1)C3(C=CC=CO3)C=N2
6	C1COC2(CO1)C=CC=CO2
7	C1C2=C(C=C(C=C2)[N+](=O)[O-])NC13C=CC=CO3
8	C1=CC2=NC3(C=CC=CO3)N=C2C=C1
9	CC\ 1(C2=CC=CC=C2N(/C1=C/C=C/C=C(C=C(C3=O)OC)[N+](=O)[O-])CCO)C
10	CCOC(=O)CCCN1C2=CC=CC=C2C([C@@]13C=CC4=C(O3)C(=CC(=C4)[N+](=O)[O-])OC)(C)C
11	C1C2=CC=CC=C2NC13C=CC=CO3
**Acyl hydra-zone**	**SMILES**
1	CCN(CC)C1=CC=C(C=C1)/C=N/NC(=O)C2=CC=C(C=C2)N
2	CC(C)(C)OC1=CC=C(C=C1)/C=N/NC(=O)C2=CC=C(C=C2)O
3	CC1=CC=C(C=C1)C(=O)N/N=C/C2=C(C(=NC=C2CO)C)O
4	C1=CC=C(C(=C1)/C=N/NC(=O)C2=CC(=CC=C2)F)O
5	CC1=NC=C(C(=C1O)/C=N/NC(=O)C2=CC=C(C=C2)[N+](=O)[O-])CO
6	CC1=NC=C(C(=C1O)/C=N/NC(=O)C2=CC=C(C=C2)N)CO
7	CC1=NC=C(C(=C1O)/C=N/NC(=O)C2=CC=CO2)CO
8	CC1=NC=C(C(=C1O)/C=N/NC(=O)C2=CC=CS2)CO
9	CC1=NC=C(C(=C1O)/C=N/NC(=O)C2=CC(=CC=C2)Cl)CO
10	CCN(CC)C1=CC=C(C=C1)/C(=N/NC(=O)C2=CC=C(C=C2)O)/C
11	CC1=NC=C(C(=C1O)/C=N/NC(=O)C2=CC=NC=C2)CO
12	C1=CC=C(C=C1)C(=O)N/N=C/C2=CC=CC=C2O
13	CC1=NC=C(C(=C1O)/C=N/NC(=O)C2=CC=CC=C2)CO
14	CC1=NC=C(C(=C1O)/C=N/NC(=O)C2=CC=C(C=C2)OC)CO
15	CC1=NC=C(C(=C1O)/C=N/NC(=O)C2=CC(=CC=C2)F)CO
16	CCN(CC)C1=CC=C(C=C1)/C=N/NC(=O)C2=CC=CC=C2
17	C1=CC=C2C(=C1)C=CC(=C2/C=N/NC(=O)C3=CC=CO3)O
18	C1=CC=C(C(=C1)/C=N/NC(=O)C2=CC=CS2)O
19	CCN(CC)C1=CC(=C(C=C1)/C=N/NC(=O)C2=CC=C(C=C2)O)O
20	CC(C)(C)C1=CC=C(C=C1)C(=O)N/N=C/C2=CC=CC=C2O
21	CC1=NC=C(C(=C1O)/C=N/NC(=O)C2=CC=C(C=C2)C(C)(C)C)CO
22	CC1=NC=C(C(=C1O)/C=N/NC(=O)C2=CC(=CC=C2)Br)CO

Continued on next page

Table S2: List of photoswitch SMILES extracted from the PubChem repository[23] (Continued)

23	CCN(CC)C1=CC=C(C=C1)/C=N/NC(=O)C2=CC=C(C=C2)F
24	C1=CC=C(C(=C1)/C=N/NC(=O)C2=CC=C(C=C2)[N+](=O)[O-])O
25	C1=CC=C2C(=C1)C=CC(=C2/C=N/NC(=O)C3=CC=C(C=C3)[N+](=O)[O-])O
26	C1=CC=C(C(=C1)/C=N/NC(=O)C2=CC=C(C=C2)N)O
27	C1=CC=C2C(=C1)C=CC(=C2/C=N/NC(=O)C3=CC=C(C=C3)N)O
28	C1=CC=C2C(=C1)C=CC(=C2/C=N/NC(=O)C3=CC(=CC=C3)F)O
29	CC(=O)N/N=C/C1=CC=CC=C1O
30	CC1=CC=C(C=C1)C(=O)N/N=C/C2=CC=CC=C2O
31	C1=CC=C(C(=C1)/C=N/NC(=O)C2=CC(=CC=C2)Cl)O
32	C1=CC=C(C(=C1)/C=N/NC(=O)C2=CC=CO2)O
33	CC1=NC=C(C(=C1O)/C=N/NC(=O)C2=CC=C(C=C2)O)CO
34	CC1=NC=C(C(=C1O)/C=N/NC(=O)C)CO
35	C1=CC=C2C(=C1)C=CC(=C2/C=N/NC(=O)C3=CC=C(C=C3)O)O
36	C1=CC=C(C(=C1)/C=N/NC(=O)C2=CC(=CC=C2)Br)O
37	COC1=CC=C(C=C1)C(=O)N/N=C/C2=CC=CC=C2O
38	CC(=O)N/N=C/C1=C(C=CC2=CC=CC=C21)O
39	C1=CC=C(C(=C1)/C=N/NC(=O)C2=CC=C(C=C2)O)O
40	COC1=CC=C(C=C1)C(=O)N/N=C/C2=C(C=CC3=CC=CC=C32)O
**Indigo and indoxyl**	**SMILES**
1	C1=CC=C2C(=C1)C(=C(N2)C3=NC4=CC=CC=C4C3=O)O
2	C1CCC2(C(C1)N3C4=C(C(=CC=C4)S2)C(=C3C5=NC6=CC=CC=C6C5=O)O)S
3	C1=CC=C2C(=C1)C(=O)C(=N2)C3=C(C4=C5N3OS(=O)(=O)OS(=O)(=O)OC4=CC=C5)O
4	C1=CC=C2C(=C1)C(=O)/C(=C/C(=O)C4=CC=CC=C4N3C(=O)C5=CC(=CC(=C5)[N+](=O)[O-])[N+](=O)[O-])/N2C(=O)C6=CC(=CC(=C6)[N+](=O)[O-])[N+](=O)[O-]
5	C[C-]1C(=C(C(=C1C)C)C)C.C1=CC=C2C(=C1)C(=O)/C(=C/3\C(=O)C4=CC=CC=C4N3)/N2.[Cl-].[Cr+2]
6	C=C1C(=O)C2=CC=CC=C2N1.C1C(=O)C2=CC=CC=C2N1
7	C1C2=CC=CC=C2N=C1C3=C(C4=CC=CC=C4N3)O
8	C1=CC=C2C(=C1)C(=C(N2)C3=C(C4=CC=CC=C4N3)[O-])[O-].[Na+].[Na+]
9	C1=CC(=CC=C1C2=CC=C(C=C2)N=NC3=C(C4=C(C=C(C=C4C=C3S(=O)(=O)O)S(=O)(=O)O)N)O)N=NC5=C(C6=C(C=C(C=C6C=C5S(=O)(=O)O)S(=O)(=O)O)N)O.[Na+]
10	C1=CC=C2C(=C1)C(=CC(=C2O)N=NC3=C4C=CC(=CC4=C(C=C3)N=NC5=C6C=CC(=CC6=C(C=C5)N=NC7=CC=C(C=C7)N)S(=O)(=O)[O-])S(=O)(=O)[O-])S(=O)(=O)[O-].[Na+].[Na+].[Na+]
11	C1=CC=C2C(=C1)C(=C(N2)O)C3=NC4=CC=CC=C4C3=O
12	C1=C(C=C(C2=C1C(=C(N2)C3=NC4=C(C3=O)C=C(C=C4S(=O)(=O)O)S(=O)(=O)O)O)S(=O)(=O)O)S(=O)(=O)O
13	C1=CC(=CC=C1C2=C(C(=O)C3=C(C=C(C=C3O2)O)O)O)O
14	CCN(CC1=CC(=CC=C1)S(=O)(=O)[O-])C2=CC=C(C=C2)C(=C3C=CC(=[N+](CC)CC4=CC(=CC=C4)S(=O)(=O)[O-])C=C3)C5=CC=CC=C5Cl.[Na+]

Continued on next page

Table S2: List of photoswitch SMILES extracted from the PubChem repository[23] (Continued)

15	CCOC1=CC2=C(C=C1)C(=C(S2)C3=C(C4=C(S3)C=C(C=C4)OCC)OS(=O)(=O)[O-])OS(=O)(=O)[O-].[Na+].[Na+]
16	CCN(CC1=CC(=CC=C1)S(=O)(=O)O)C2=CC=C(C=C2)C(=C3C=CC(=[N+](CC)CC4=CC(=CC=C4)S(=O)(=O)O)C=C3)C5=CC=CC=C5Cl
17	C1=CC=C2C(=C1)C(=O)/C(=C/C(=O)C4=CC=CC=C4S3)/S2
18	CCCCCC[C@@H](C)OC1=C(C=C2C(=C1)S/C(=C/3\C(=O)C4=CC(=C(C=C4S3)O[C@H](C)CCCCCC)Cl)/C2=O)Cl
19	CCCCCC.C[C-]1C(=C(C(=C1C)C)C)C.C[C-]1C(=C(C(=C1C)C)C)C.C1=CC=C2C(=C1)C(=C(S2)C3=C(C4=CC=CC=C4S3)[O-])[O-].C1=CC=C2C(=C1)C(=C(S2)C3=C(C4=CC=CC=C4S3)[O-])[O-].[Cr+3].[Cr+3]
20	CC1=CC(=CC2=C1C(=O)/C(=C/C(=O)C4=C(S3)C=C(C=C4C)Cl)/S2)Cl
21	C1=CC=C2C(=C1)C=CC3=C2C(=O)/C(=C/C(=O)C5=C(S4)C=CC6=CC=CC=C65)/S3
22	C1=CC2=C(C(=C1)Cl)C3=C(C=C2)C(=C(S3)C4=NC5=C(C4=O)C=C(C=C5)Br)O
23	CC1=CC(=C(C2=C1S/C(=C/3\ C(=O)C4=C(S3)C(=CC(=C4C)Cl)C)/C2=O)C)Cl
24	CCOC1=CC2=C(C=C1)C(=O)/C(=C/C(=O)C4=C(S3)C=C(C=C4)OCC)/S2

1.3 C: Add 5–6/membered rings

Subsequently, we manually generated a set of 5/6-membered ring molecules to introduce diversity in the aromatic chemical environments within our database. Starting from the ground up, we employed a combinatorial approach utilizing the SMILES representations outlined in Table S3. This process yielded an extensive pool of potential molecules (1,000,000 structures). Subsequently, molecules that did not exhibit an aromatic cycle and lacked photochromic units were excluded. After eliminating duplicates, structures that failed to converge at the DFTB-D3/3ob level, and those showing significantly distorted geometries with broken covalent bonds, we isolated 3,165 molecules.

Table S3: List of SMILES used to generate the 5– and 6– membered aromatic rings

**Ring**	**SMILES**
1	c1cccc1
2	c1nccc1
3	c1occc1
4	c1sccc1
5	c1nncc1

Continued on next page

Table S3: List of SMILES used to generate the 5– and 6– membered aromatic rings (Continued)

6	c1ncnc1
7	c1oncc1
8	c1ocnc1
9	c1sncc1
10	c1scnc1
11	c1ococ1
12	c1ocsc1
13	c1nncn1
14	c1nnnc1
15	c1nonc1
16	c1scnn1
17	c1nsnc1
18	c1nsnc1
19	c1nnnn1
20	c1ccccc1
21	c1ccccn1
22	c1ccccs1
23	c1cccco1
24	c1cccnn1
25	c1ncccn1
26	c1cnccn1
27	c1concc1
28	c1cocnc1
29	c1coccn1
30	c1cnccs1
31	c1cccns1
32	c1nnncc1
33	c1ncncn1
**FGs**	**SMILES**
1	(O)
2	(=O)
3	(OC)
4	(OOC)
5	(C(=O)O)
6	(C(OC)OC)
7	(S)
8	(=S)
9	(SC)
10	(C(=S)O)
11	(C(=S)OC)

Continued on next page

Table S3: List of SMILES used to generate the 5– and 6– membered aromatic rings (Continued)

12	(C(=O)S)
13	(C(=O)SC)
14	(N)
15	(NC)
16	(N(C)(C))
17	(NN=C(C)(C))
18	(NN)
19	(N=N(C)(C))
20	([N+](=O)[O-])
21	(C(=O)N)
22	(NC(=O)C)
23	(C)
24	(=C)
25	(=C)
26	(C=C)
27	(C=CC=C)
28	(C#C)
29	(C#N)
30	(C(F)(F)(F))
31	(F)

1.4 D: Add non–covalent interactions

Finally, to account for non–covalent interactions between different organic fragments, we resorted to the NCI Atlas database.[24–26] We extracted the whole dimers containing only the desired atom types from the various sub–databases available on the database's web server. Below is the list of the sub–databases considered:

- D1200 - London dispersion in an extended chemical space
- SH250×10 - sigma-hole interactions
- R739×5 - Repulsive contacts in an extended chemical space
- HB300SPX×10 - Hydrogen bonding extended to S, P and halogens
- HB375×10 - Hydrogen bonding in organic molecules

After filtering duplicates and structures that failed during optimization at the DFTB-D3/3ob level, a total of 633 complexes were obtained.

1.5 E/F: Conformational diversity

Having ensured chemical diversity, we next focused on adding conformational diversity to the database. To do so, we performed DFTB molecular dynamic trajectories (up to 5 ps) at 300 and 600K on the 7,869 entries. From each trajectory, the most diverse conformations were selected using the FPS algorithm on the SLATM representation. Depending on the size and flexibility of each structure, between 5 and 20 conformations were selected, giving a total of 67,505 out–of–equilibrium structures. Here, empirical choices were made to best balance not only molecular size and flexibility (*i.e.*, more conformers were selected for larger and more flexible systems, fewer for smaller and more rigid systems) but also to assure an abundance of different functional groups.

2 Accuracy of the Machine Learning Potential

Training of a Local Kernel Regression

The Local Kernel Regression is an in–house code developed by Fabregat *et al.*[27] and freely available at `https://github.com/lcmd-epfl/Local_Kernel_Regression`. The main protocol to train an LKR–OMP model is explained in the README.md file. You just need to provide two specific files:

1. A file containing all your structures concatenated in a xyz format (with units in Angstrom). The Atomic Simulation Environment (ASE) for instance can easily do it in python.

2. A file containing all the concatenated energies related to each structure listed in the previous xyz file, with the units in kcal/mol. As these are the target of the model, it is on the choice of the user which type of energy (DFT, Δ, ...) to consider. In this study, we consider the Δ correction between DFTB3/3ob and PBE0–D3/def2svp.

The type of descriptor can be easily adapted in the python file 1-gen_reps.py and can be whatever descriptor available in the python librairies (FCHL, SLATM, ...). The hyper-parameters related to the training can be adjusted in the python files 4-gen_diffs.py and 5-gen_projections.py.

Table S4: MAE predicted for different functional groups using DFTB and DFTB+LKR corrections with respect to PBE0-D3 references using the LKR–OMP with the best 1,000 environments.

Functional groups	DFTB MAE (kcal/mol)	DFTB+LKR MAE (kcal/mol)
Alcohol	6.70	1.43
Ketone	7.69	1.46
Aldehyde	8.73	1.22
Ester	7.35	1.44
Peroxides	6.59	1.52
Ether	7.94	1.46
Carboxylic acid	5.44	1.35
Thiol	7.56	1.41
Carbo–thioester	5.05	1.11
Thiocarbonyl	6.82	1.43
Thioamide	8.99	1.79
Thio–ether	7.59	1.51
Amide	7.77	1.49
Enamine	8.00	1.72
1ary amine	9.43	1.43
2ary amine	8.71	1.49
3ary amine	12.05	1.84
Azo	9.06	1.53
Hydrazine	10.78	1.87
Hydrazone	9.00	1.78
Iminium	10.99	2.21
Cyano	27.59	1.65
Nitro	10.85	2.06
6mem ring	8.21	1.40
6mem hetero	9.25	1.39
5mem hetero	8.22	1.45
Fluorine	8.36	1.60
Only alkyl	12.67	1.21

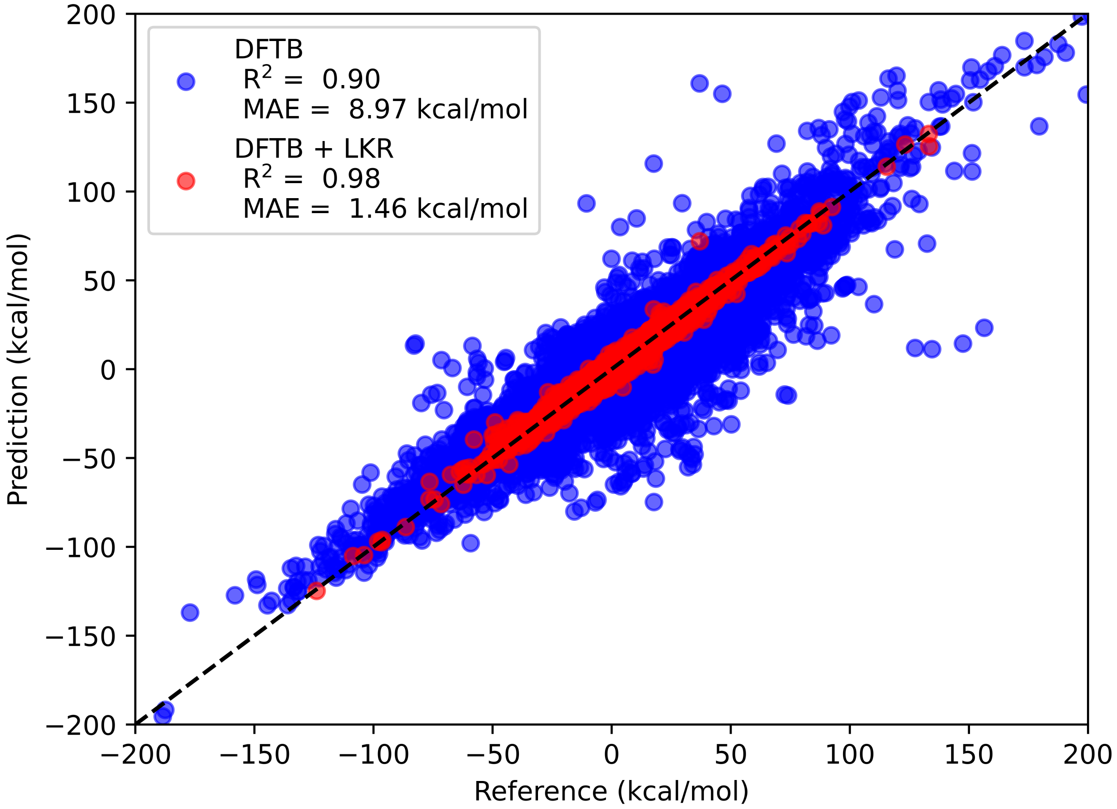

Figure S1: Parity plot of the test set for the DFTB and DFTB+LKR predictions. The respective MAE and R^2 are also decpited.

3 Identify the local atomic contribution in LKR–OMP computations

To generate scalar fields, we computed the DFT correction for each atom of for the systems shown in Fig. S2. Subsequently, we convoluted the DFT corrections with the atomic positions and applied a Gaussian filter with a width of 1 Å for each system. This operation can be performed using Python in conjunction with the scipy library. Consequently, we obtained the DFT correction for the entire molecules, factoring in all DFT corrections from neighboring atoms. The final result is saved in the cubefile format and can be visualized using the VMD software, where each isosurface corresponds to a level of the scalar field, quantified in kcal/mol.Å. Within each structure, we identified distinct regions of space

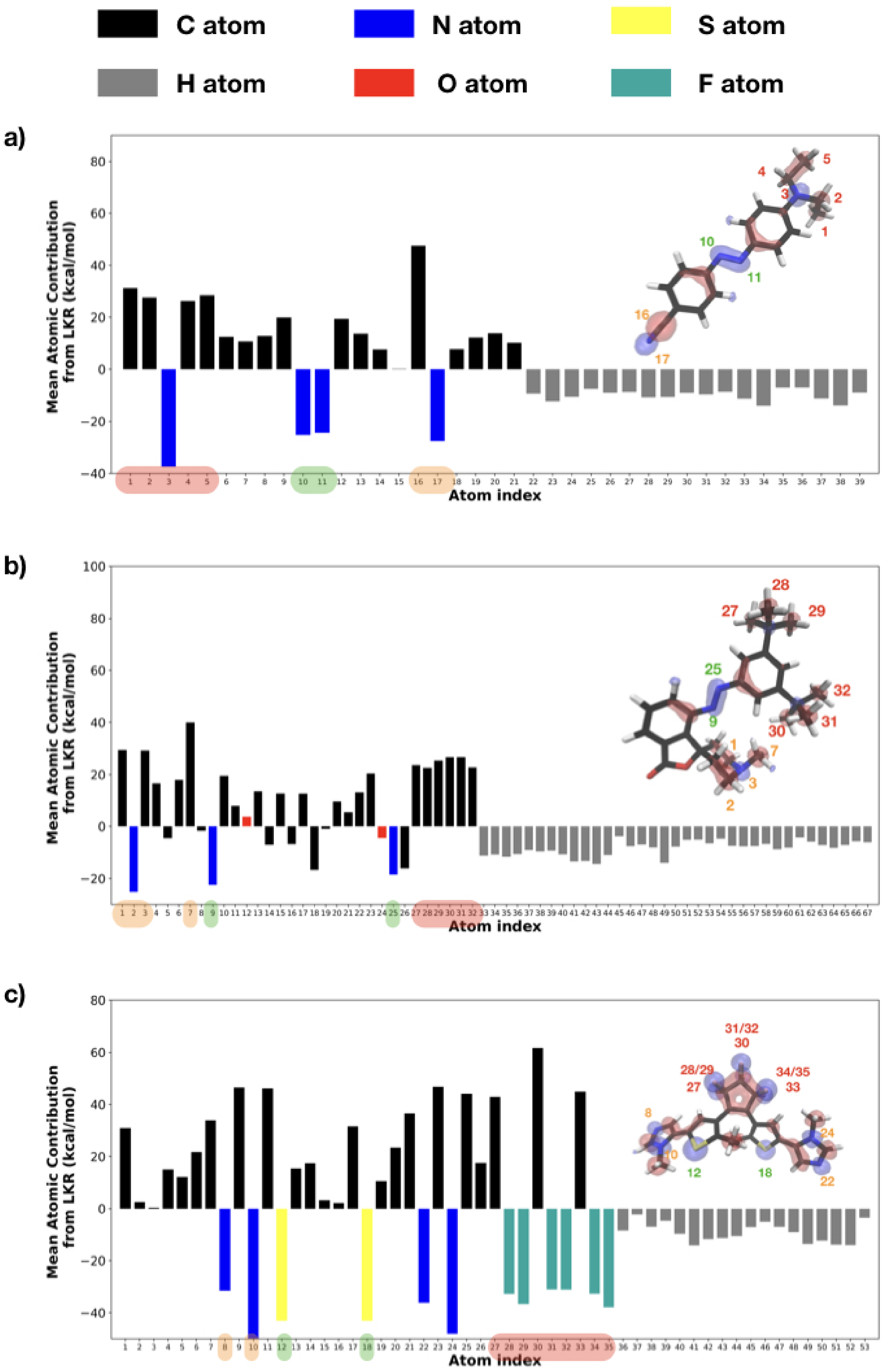

Figure S2: Histograms representing the mean atomic contribution to the LKR–OMP corrections for structures used in section 4.2 in the main text.

where the LKR–OMP correction exerted a notable influence on the energetic correction of the structure. These regions were annotated with atomic numbers and are correspondingly highlighted in the x-axis of each histogram in Fig. S2.

4 Convergence of the Hamiltonian Reservoir Replica Exchange simulations

4.1 *(E)*–PS1

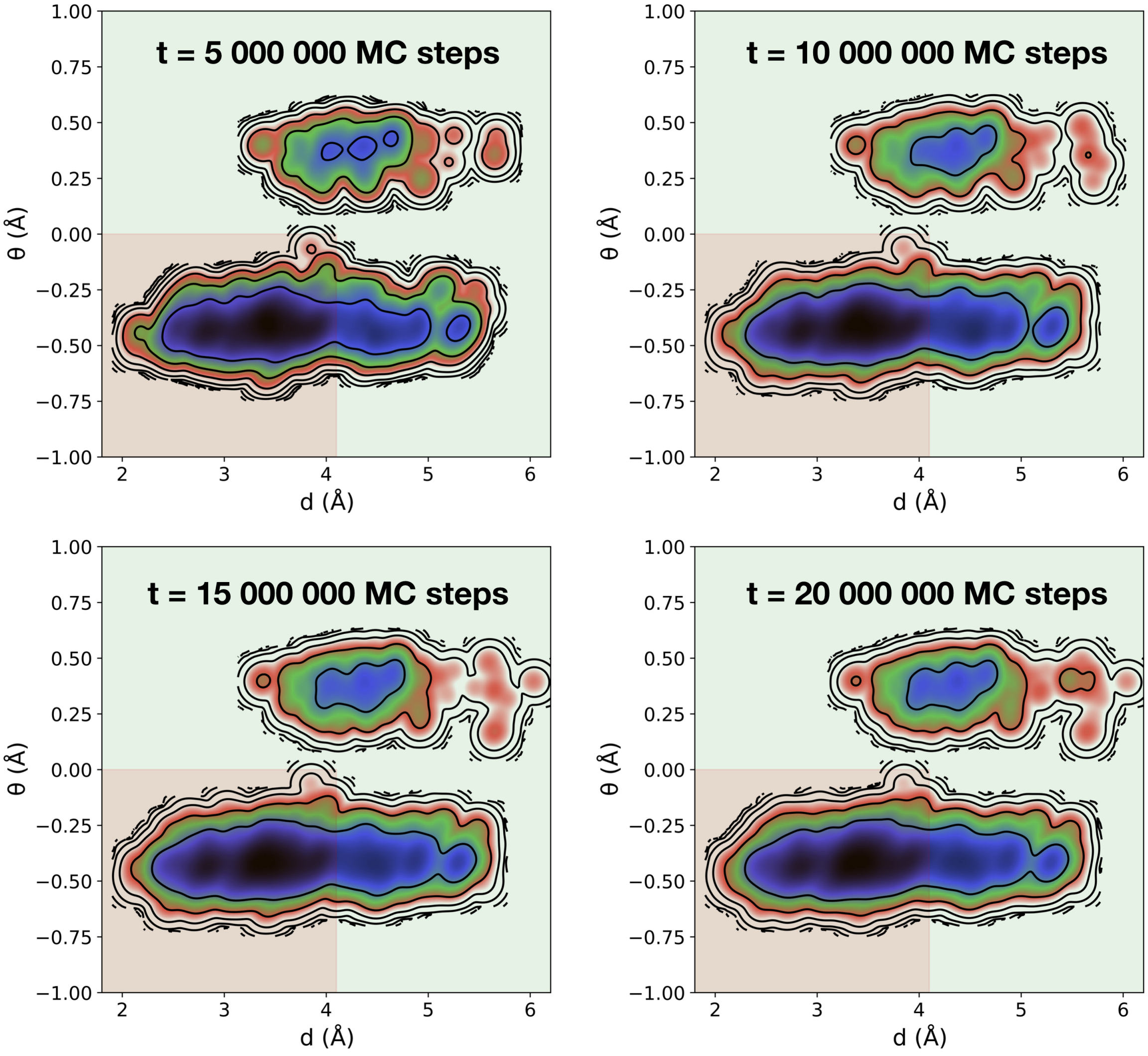

Figure S3: Convergence of the free energy profile for *(E)*–PS1

4.2 *(Z)*–PS1

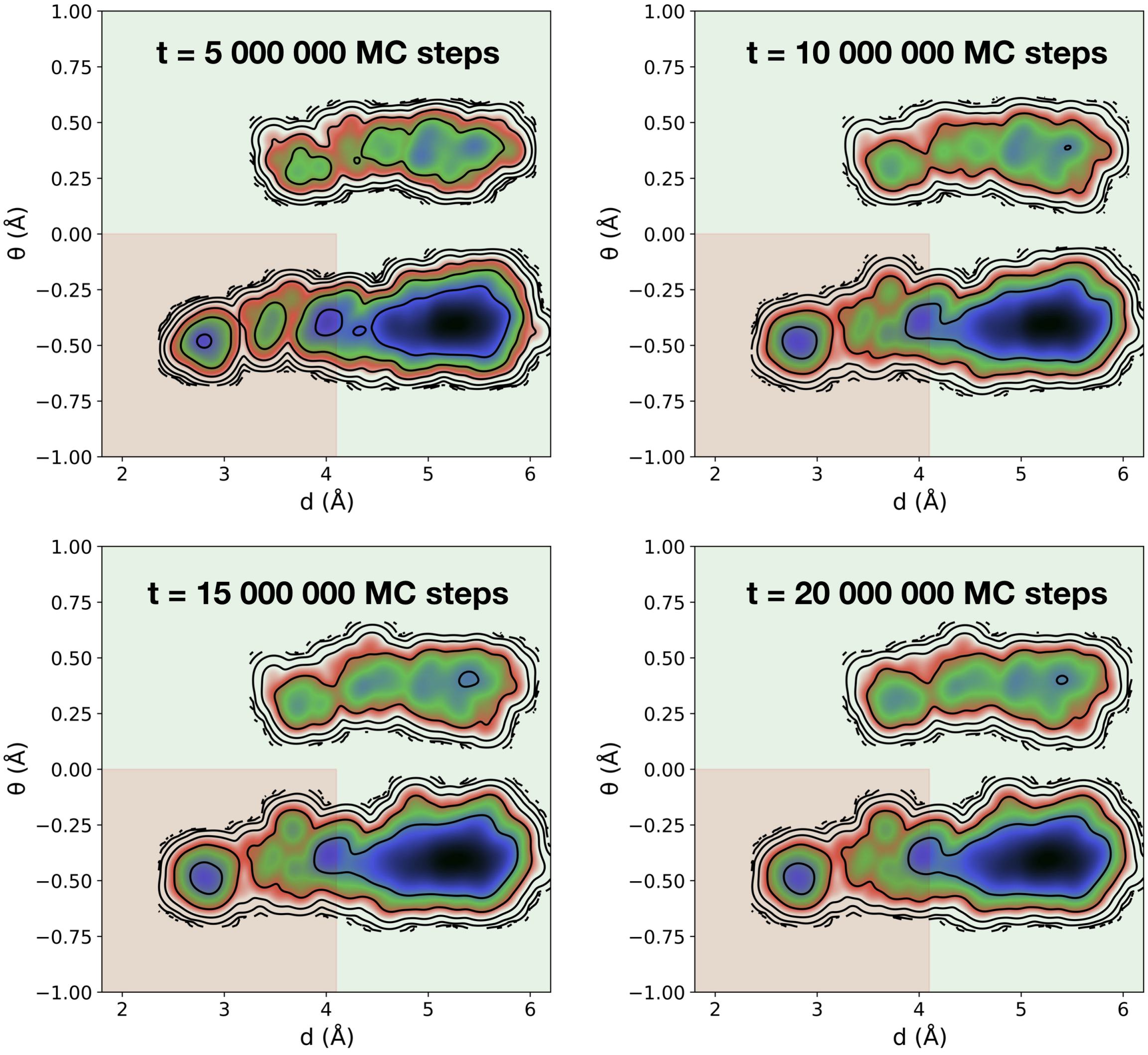

Figure S4: Convergence of the free energy profile for *(Z)*–PS1

4.3 *(E)*–PS2

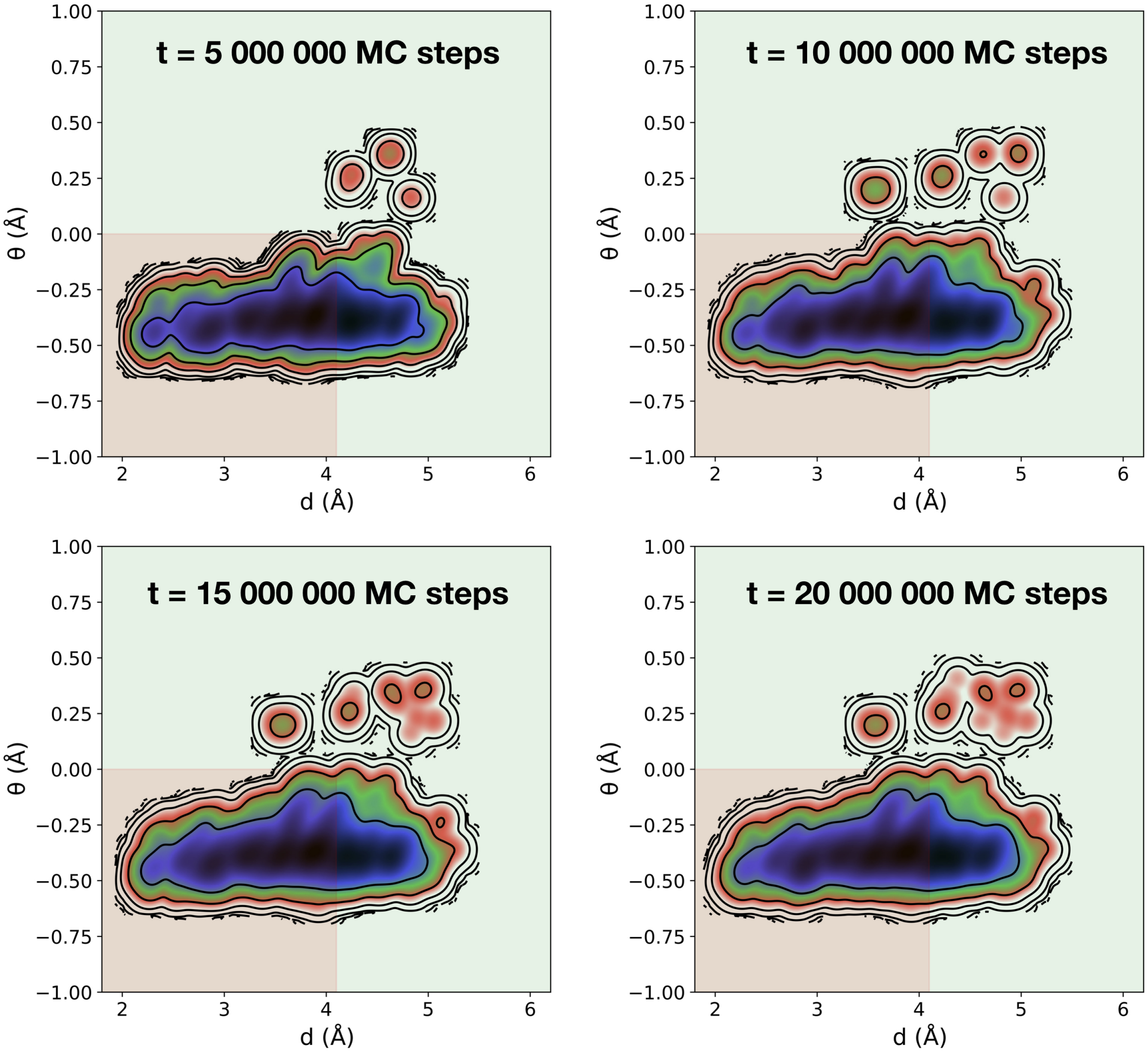

Figure S5: Convergence of the free energy profile for *(E)*–PS2

4.4 *(Z)*–PS2

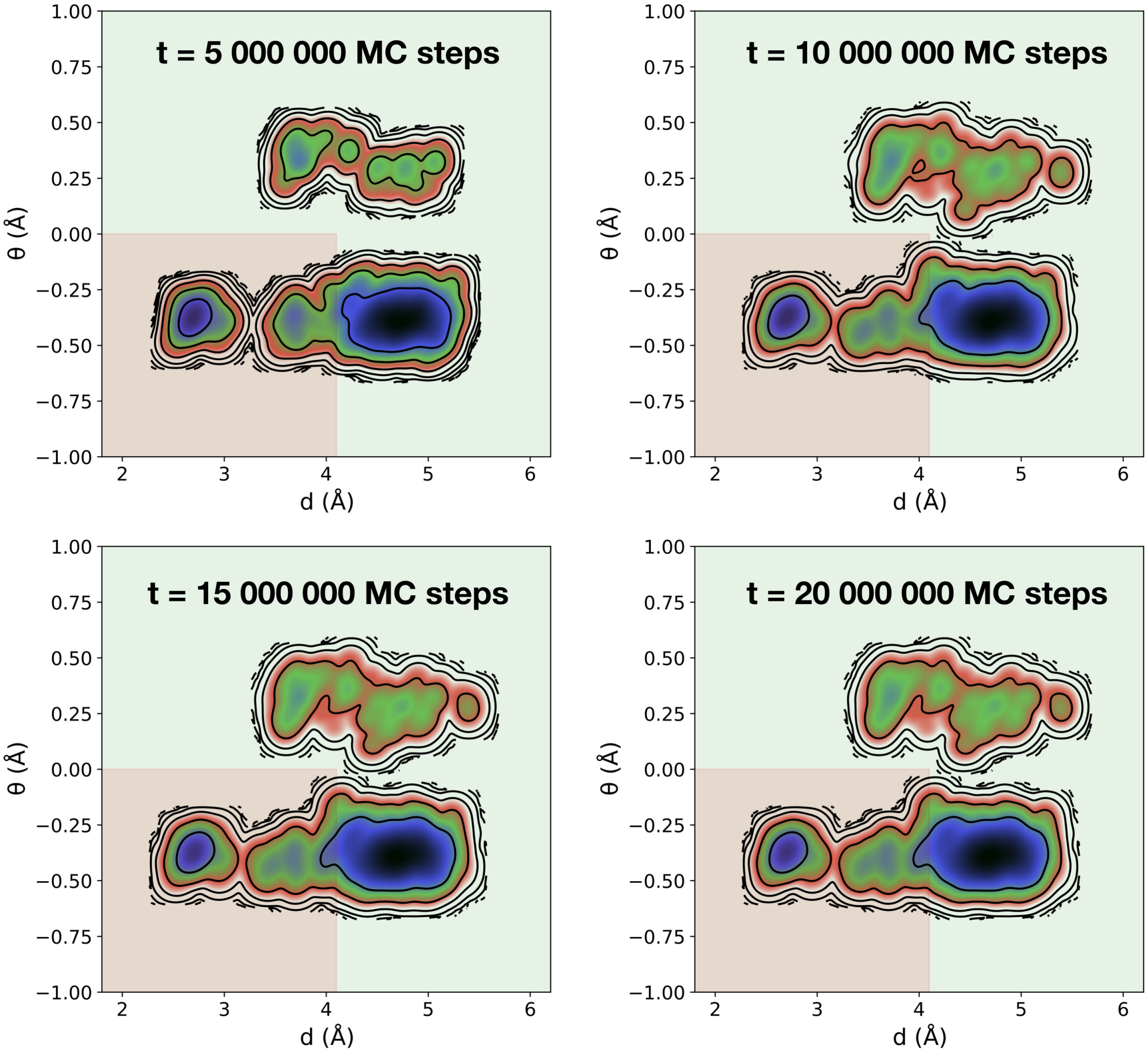

Figure S6: Convergence of the free energy profile for *(Z)*–PS2

References

(1) Gallarati, S.; van Gerwen, P.; Laplaza, R.; Vela, S.; Fabrizio, A.; Corminboeuf, C. OSCAR: An Extensive Repository of Chemically and Functionally Diverse Organocatalysts. *Chem. Sci.* **2022**, *13*, 13782–13794.

(2) De Bo, G.; Leigh, D. A.; McTernan, C. T.; Wang, S. A Complementary Pair of Enantioselective Switchable Organocatalysts. *Chem. Sci.* **2017**, *8*, 7077–7081.

(3) Nojiri, A.; Kumagai, N.; Shibasaki, M. In Situ Manipulation of Catalyst Performance via the Photocontrolled Aggregation/Dissociation State of the Catalyst. *Chem. Commun.* **2013**, *49*, 4628–4630.

(4) Stoll, R. S.; Peters, M. V.; Kuhn, A.; Heiles, S.; Goddard, R.; Bühl, M.; Thiele, C. M.; Hecht, S. Photoswitchable Catalysts: Correlating Structure and Conformational Dynamics with Reactivity by a Combined Experimental and Computational Approach. *J. Am. Chem. Soc.* **2009**, *131*, 357–367.

(5) Imahori, T.; Yamaguchi, R.; Kurihara, S. Azobenzene-Tethered Bis (Trityl Alcohol) as a Photoswitchable Cooperative Acid Catalyst for Morita–Baylis–Hillman Reactions. *Chem. Eur. J.* **2012**, *18*, 10802–10807.

(6) Neilson, B. M.; Bielawski, C. W. Photoswitchable Organocatalysis: Using Light To Modulate the Catalytic Activities of N-Heterocyclic Carbenes. *J. Am. Chem. Soc.* **2012**, *134*, 12693–12699.

(7) Chen, C.-T.; Tsai, C.-C.; Tsou, P.-K.; Huang, G.-T.; Yu, C.-H. Enantiodivergent Steglich Rearrangement of O-Carboxylazlactones Catalyzed by a Chirality Switchable Helicene Containing a 4-Aminopyridine Unit. *Chem. Sci.* **2017**, *8*, 524–529.

(8) Wang, J.; Feringa, B. L. Dynamic Control of Chiral Space in a Catalytic Asymmetric Reaction Using a Molecular Motor. *Science* **2011**, *331*, 1429–1432.

(9) Eisenreich, F.; Kathan, M.; Dallmann, A.; Ihrig, S. P.; Schwaar, T.; Schmidt, B. M.; Hecht, S. A Photoswitchable Catalyst System for Remote-Controlled (Co) Polymerization In Situ. *Nat. Catal.* **2018**, *1*, 516–522.

(10) Iida, H.; Umebayashi, N.; Yashima, E. Photoswitchable Organocatalysis in Acylation of Alcohol Using Dithienylethene-Linked Azoles. *Tetrahedron* **2013**, *69*, 11064–11069.

(11) Osorio-Planes, L.; Rodriguez-Escrich, C.; Pericàs, M. A. Photoswitchable Thioureas for the External Manipulation of Catalytic Activity. *Org. Lett.* **2014**, *16*, 1704–1707.

(12) Kathan, M.; Eisenreich, F.; Jurissek, C.; Dallmann, A.; Gurke, J.; Hecht, S. Light-Driven Molecular Trap Enables Bidirectional Manipulation of Dynamic Covalent Systems. *Nat. Chem.* **2018**, *10*, 1031–1036.

(13) Vlatković, M.; Bernardi, L.; Otten, E.; Feringa, B. L. Dual Stereocontrol Over the Henry Reaction Using a Light–And Heat-Triggered Organocatalyst. *Chem. Commun.* **2014**, *50*, 7773–7775.

(14) Pizzolato, S. F.; Collins, B. S.; van Leeuwen, T.; Feringa, B. L. Bifunctional Molecular Photoswitches Based on Overcrowded Alkenes for Dynamic Control of Catalytic Activity in Michael Addition Reactions. *Chem. Eur. J.* **2017**, *23*, 6174–6184.

(15) Wilson, D.; Branda, N. R. Turning “On” and “Off” a Pyridoxal 5-Phosphate Mimic Using Light. *Angew. Chem., Int. Ed.* **2012**, *51*, 5431–5434.

(16) Dai, Z.; Cui, Y.; Chen, C.; Wu, J. Photoswitchable Ring-Opening Polymerization of Lactide Catalyzed by Azobenzene-Based Thiourea. *Chem. Commun.* **2016**, *52*, 8826–8829.

(17) Thawani, A. R.; Griffiths, R.-R.; Jamasb, A.; Bourached, A.; Jones, P.; McCorkindale, W.; Aldrick, A. A.; Lee, A. A. The Photoswitch Dataset: A Molecular Machine Learning Benchmark for the Advancement of Synthetic Chemistry. *arXiv preprint arXiv:2008.03226* **2020**.

(18) Huang, B.; von Lilienfeld, O. A. Quantum Machine Learning Using Atom-in-Molecule-Based Fragments Selected on the Fly. *Nat. Chem.* **2020**, *12*, 945–951.

(19) Vela, S.; Laplaza, R.; Cho, Y.; Corminboeuf, C. cell2mol: Encoding Chemistry To Interpret Crystallographic Data. *Npj Comput. Mater.* **2022**, *8*, 188.

(20) Groom, C. R.; Bruno, I. J.; Lightfoot, M. P.; Ward, S. C. The Cambridge Structural Database. *Acta. Crystallogr. B. Struct. Sci. Cryst. Eng. Mater.* **2016**, *72*, 171–179.

(21) Landrum, G. et al. RDKit: A Software Suite for Cheminformatics, Computational Chemistry, and Predictive Modeling. *Greg Landrum* **2013**.

(22) Jensen, J. H. xyz2mol. *GitHub repository* **2020**.

(23) Kim, S.; Chen, J.; Cheng, T.; Gindulyte, A.; He, J.; He, S.; Li, Q.; Shoemaker, B. A.; Thiessen, P. A.; Yu, B.; Zaslavsky, L.; Zhang, J.; Bolton, E. E. PubChem 2019 Update: Improved Access to Chemical Data. *Nucleic Acids Res. Spec. Publ.* **2019**, *47*, D1102–D1109.

(24) Řezáč, J. Non-Covalent Interactions Atlas Benchmark Data Sets: Hydrogen Bonding. *J. Chem. Theory. Comput.* **2020**, *16*, 2355–2368.

(25) Řezáč, J. Non-Covalent Interactions Atlas Benchmark Data Sets 5: London Dispersion in an Extended Chemical Space. *Phys. Chem. Chem. Phys.* **2022**, *24*, 14780–14793.

(26) Řezáč, J. Non-Covalent Interactions Atlas Benchmark Data Sets 2: Hydrogen Bonding in an Extended Chemical Space. *J. Chem. Theory. Comput.* **2020**, *16*, 6305–6316.

(27) Fabregat, R.; Fabrizio, A.; Engel, E. A.; Meyer, B.; Juraskova, V.; Ceriotti, M.; Corminboeuf, C. Local Kernel Regression and Neural Network Approaches to the Conformational Landscapes of Oligopeptides. *J. Chem. Theory. Comput.* **2022**, *18*, 1467–1479.
